# A comparison of DNA methylation in newborn blood samples from infants with and without orofacial clefts

**DOI:** 10.1186/s13148-019-0638-9

**Published:** 2019-03-04

**Authors:** Zongli Xu, Rolv T. Lie, Allen J. Wilcox, Ola Didrik Saugstad, Jack A. Taylor

**Affiliations:** 1 Epidemiology Branch, National Institute of Environmental Health Sciences, NIH, Research Triangle Park, NC USA; 2 Laboratory of Molecular Carcinogenesis, National Institute of Environmental Health Sciences, NIH, P.O. Box 12233, Mail Drop A3-05, Research Triangle Park, NC 27709 USA; 3 Department of Global Public Health and Primary Care, University of Bergen, Bergen, Norway; 4 Centre for Fertility and Health, Norwegian Institute of Public Health, Oslo, Norway; 5 Department of Pediatric Research, Oslo University Hospital, Oslo, Norway; 6 Department of Clinical Medicine, University of Oslo, Oslo, Norway

**Keywords:** DNA methylation, Cleft lip, Cleft palate, Blood, Newborns

## Abstract

**Background:**

Isolated orofacial clefts are among the most common congenital birth defects. Although the underlying biological mechanisms remain largely unknown, clefts are thought to be complex disorders influenced by genetic, environmental, and potentially epigenetic factors.

**Methods:**

In blood samples from 2- to 3-day-old infants (*n* = 747) collected in a nationwide population-based study of orofacial clefts in Norway, we measured DNA methylation profiles for more than 450,000 CpGs and then conducted epigenome-wide association analyses (EWAS). We tested methylation profile difference at each CpG between controls (*n* = 436) and each of the cleft subtypes (92 cleft lip only, CLO; 84 cleft palate only, CPO; 132 cleft lip and palate, CLP). We also compared controls to various combinations of case groups and compared case subtypes to each other. Finally, using the EWAS results, we searched for larger differentially methylated regions (DMRs) associated with orofacial clefts.

**Results:**

In EWAS comparing controls to individual cleft subtypes, we found no significant associations at a Bonferroni *P* value threshold of 10^−7^. After pooling case groups, we found two significantly differentially methylated CpGs: cg09696939 near gene BICC1 is associated with CLO+CLP (*P* = 9.58 × 10^−8^); cg26985354 in gene CLASRP is associated with CPO+CLP (*P* = 7.38 × 10^−8^). In DMR analysis, we identified a total of 56 significant regions when comparing controls to individual cleft subtypes (10 for CLO, 6 for CPO, 41 for CLP). Only one DMR is shared among the three cleft groups. In combined case group analysis, we found 26 DMRs for CLP+CLO, 31 for CLP+CPO, and 37 when all subtypes are combined. Finally, in case-case comparisons of subtypes, we identified 10 DMRs when comparing CLP to CPO, 9 in CLP compared to CLO, and 13 in CLP compared to CPO.

**Conclusions:**

We identified two individual CpGs and multiple DMRs that differ between controls and cleft case subtypes. Although we find some evidence for the possible role of DNA methylation in etiology of orofacial clefts, our study does not support previous reports of widespread differences in blood DNA methylation between babies with and without facial clefts.

**Electronic supplementary material:**

The online version of this article (10.1186/s13148-019-0638-9) contains supplementary material, which is available to authorized users.

## Background

Orofacial clefts are among the most common congenital birth defects. Approximately 250,000 babies are born with these defects each year worldwide (http://cleft.org.uk/), and the prevalence varies by ethnicity, sex [1], and socioeconomic status [2]. Orofacial clefts, which include three distinct subgroups, cleft lip only (CLO), cleft lip and palate (CLP), and cleft palate only (CPO), result from fusion failures in weeks 5 (fusion of lip) and 9 (fusion of palate) from date of conception. An orofacial cleft that is left unrepaired can have a substantial adverse impact on the health, quality of life, and psychosocial well-being of the child. Full repair of clefts entails surgery, dental treatment, and speech therapy, imposing financial and emotional burden on families [3].

Approximately 70% of orofacial clefts are non-syndromic [1], and the underlying causes of these isolated cases are complex and remain largely unknown. Although familial recurrence risk estimates and segregation analysis [4–6] suggest there is strong evidence for a genetic component, environmental factors like smoking, vitamin intake, and alcohol intake may also play an important role [7–9]. To date, whole genome linkage and SNPs identified through genome-wide association studies can explain only a small percentage of the disease variation [1, 10]. Several recent studies [8, 11–13] support a multifactorial threshold model of inheritance in which small genetic risk factors may interact with environmental factors.

Epigenetic changes, in particular DNA methylation, can mediate both genetic and environmental factors and may advance the understanding of the molecular pathogenesis of orofacial clefts [14]. Several recent large studies [15–18] show that maternal exposure to putative cleft risk factors, such as tobacco smoking, obesity, and folate deficiency, can affect methylation profiles of blood DNA in newborns and infants. Alvizi et al. [19] performed an epigenome-wide association study of blood DNA from 67 cases and 59 controls using Illumina 450K array and reported 578 differentially methylation CpGs. In a Mendelian randomization analysis in the Avon Longitudinal Study of Parents and Children (ALSPAC) [20], Howe et al. [21] reported 21 putative CpG sites where methylation might mediate genetic allelic effects on liability to non-syndromic cleft lip/palate. Sharp et al. [22] compared DNA methylation profiles between cleft subtypes (50 samples of each subtype) and reported a total of 121 differentially methylated regions (DMRs) in pairwise comparisons using blood DNA and 15 DMRs using lip/palate DNA. Here, we report a larger epigenome-wide blood DNA methylation study from a national population-based case-control study of orofacial clefts in Norway.

## Methods

### Study samples

This study used blood samples from infants in our nationwide population-based Norway Facial Cleft Study (NCL), which has been previously described in detail [9]. Four hundred eighteen infants born with an orofacial cleft during the years 1996 to 2001 were included: 107 CLO, 167 CLP, and 144 CPO. A total of 480 control infants born without clefts were randomly selected from all live births in Norway during that time period. Heel-stick blood samples had been collected from all babies 2–3 days after delivery for phenylketonuria (PKU) testing. These samples were stored and later used for DNA methylation analysis. Demographic characteristics, mother’s environmental exposures, and nutrition intake were collected through questionnaires approximately 3 months after delivery.

Ethical approval for this study was obtained from the Norwegian Data Inspectorate, the Regional Research Ethics Committee for Western Norway, and the Institutional Review Board of the US National Institute of Environmental Health Sciences, National Institutes of Health. Parents gave consent to data and sample collection, and all data were anonymized after collection.

### DNA methylation measures

Genomic DNA was extracted from whole blood using automated equipment (Autopure LS; Gentra, Minneapolis, MN). Extracted DNA was quantified using Quant-iT™ PicoGreen dsDNA reagents (Invitrogen) and stored at − 20 °C. One microgram of DNA was bisulfite converted using the EZ-DNA Methylation kit (Zymo Research, Irvine, CA) following the manufacturer’s protocol. Samples were randomly distributed across ten 96-well plates used for laboratory processing. The Illumina HumanMethylation450 BeadChip (San Diego, CA) was used to assess DNA methylation at 485,577 CpG sites with 99% coverage of RefSeq genes and an average of 17 CpG sites per gene distributed across the promoter, 5′ untranslated region (UTR), first exon, gene body, and 3′UTR. At each CpG site on the array, the methylation level was estimated using intensity measures of probes corresponding to an unmethylated (*U*) or methylated (*M*) signal. Raw probe intensity values were extracted using R package illuminaio (version 0.18.0) [23].

### Methylation data pre-processing

Methylation raw IDAT files were preprocessed using ENmix R package [24]. Briefly, we performed background correction using the ENmix method [24]; fluorescent dye bias between Cy3 and Cy5 was adjusted with the RELIC method [25]; inter-array quantile normalization was applied on methylation intensity values to make them comparable between samples; and Infinium I and II probe-type design bias was adjusted using the RCP method [26]. The methylation level (*β* value) for each CpG site was calculated as the ratio of normalized fluorescent intensities between methylated and unmethylated alleles *β = M*/(*M + U +* 100).

We excluded 29 samples with low-quality methylation data according to the following criteria: (1) average intensity value across Illumina’s bisulfite internal control probes less than 5500, (2) more than 5% of CpG probes having low-quality data (Illumina detection *P* value > 10^−6^, read from less than 3 beads, or outlier value for the probe in the dataset), and (3) clear outliers based on visual inspection of a density plot of total intensity (*U* + *M*). We excluded one sample due to sex ambiguity. We also removed poorly performing CpG probes as follows: (1) CpGs (*n* = 23,264) with more than 5% low-quality data; (2) CpGs (*n* = 53,247) with common SNPs (minor allele frequency ≥ 0.05 in Europeans based on 1000 Genomes Project data) located at the probe’s target CpG site, or probes mapping to multiple genomic locations [27], or CpGs on X or Y chromosomes; (3) we further excluded 1488 CpGs with multiple mode distributions identified with ENmix R package. After sample and CpG probe exclusions, 407,513 CpGs and 868 samples (456 controls, 105 CLO, 167 CLP, and 140 CPO) remained for analysis.

### Statistical analysis

An unconditional logistic regression model was employed to perform epigenome-wide association analysis (EWAS). We tested for an association between DNA methylation *M* values (logit transformation of *β* values) at each CpG and a binary variable for each type of orofacial cleft (shared controls vs. each cleft type: CLO, CPO, CLP) or between cleft types (CLO vs CPO, CLP vs CLO, and CLP vs CPO). In all association tests, we adjusted for covariables including five surrogate variables (that explain 96% of variations in non-negative control-probe data), plate, gender, calendar year of baby’s birth, gestational age, and blood cell subtype composition. The proportions of five leukocyte subtypes (T cell, B cell, granulocyte, monocyte, and natural killer cell) were estimated using a method proposed by Houseman et al. [28] with reference dataset GSE35069 [29]. To correct for multiple testing, we employed the Bonferroni method and use *P* value threshold of 10^−7^ as the statistical significance cutoff in all genome-wide analyses. All statistical association analyses were performed using the R software package (version 3.4). In addition to the EWAS for individual CpG, we also used software DMRcate [30] and Comb-P [31] to detect differentially methylated regions (DMRs) based on individual CpG *P* values from the EWAS. A DMR was defined as regions containing at least two CpGs less than 1000 base pairs apart with Sidak multiple testing-corrected *P* value < 0.05.

## Results

### Sample characteristics

To focus our investigation on non-syndromic facial clefts, we excluded babies with other birth defects, resulting in 308 cases (92 CLO, 84 CPO, 132 CLP) and 436 controls in the analysis. Demographic characteristics of these infants and their mothers, as well as maternal exposures during pregnancy, are summarized in Table 1. Compared to controls, CLP cases are more likely to be male, and CPO cases are more likely to be female. These are consistent with the literature [32]. On average, infants with CLO or CPO weigh slightly less at birth (ANOVA test *P* = 1.7 × 10^−2^). Active maternal smoking during the first trimester (≥ 1 cigarette per day) is positively associated with CLO (OR = 2.5, CI = (1.6–4.0)) and CLP (OR = 1.8, CI = (1.2–2.7)), but not with CPO (OR = 1.4, CI = (0.8, 2.3)), consistent with an earlier analysis of the full dataset [7]. Mothers with less education have an elevated risk of babies with CLO (OR = 2.4, CI = (1.3–4.3)) and CLP (OR = 1.9, CI = (1.1–3.3)). Lower dietary folate during pregnancy is a risk factor for CPO (OR = 0.58, CI = (0.35–0.96)) [9]. Additional maternal characteristics were examined, such as age at delivery, body mass index (BMI), and multivitamin use, but none were associated with facial clefts.

**Table 1:** Characteristics of infants and mothers

	Control	CLO	CPO	CLP
Infants	436	92	84	132
Sex, *n* (%)				
Female	198 (45)	41 (45)	47 (56)	37 (28)
Male	238 (55)	51 (55)	37 (44)	95 (72)
Birth weight, kg	3.6 ± 0.6	3.4 ± 0.7	3.5 ± 0.6	3.6 ± 0.6
Gestational age, days	277.7 ± 11.6	274.8 ± 14.6	276.4 ± 13.8	278.6 ± 11.7
Mothers				
Age at delivery, years	29.1 ± 4.8	28.9 ± 4.6	29.2 ± 5.0	29.4 ± 5.4
Active smoker^a^, *n* (%)				
No	316 (72)	47 (51)	55 (65)	78 (59)
Yes	120 (28)	45 (49)	29 (35)	54 (41)
Alcohol use^b^, *n* (%)				
0	292 (66)	52 (56)	56 (66)	80 (61)
1–3	73 (17)	19 (21)	14 (17)	28 (21)
4–6	26 (6)	6 (7)	8 (10)	8 (6)
≥ 7	42 (10)	12 (13)	6 (7)	14 (11)
Missing	3 (1)	3 (3)	0 (0)	2 (1)
BMI (kg/m^2^)	23.5 ± 3.9	23.5 ± 3.7	23.7 ± 4.8	23.3 ± 4.1
Folic acid supplement, μg^c,d^, *n* (%)				
0	261 (60)	59 (65)	47 (56)	94 (71)
1–399	90 (21)	17 (18)	19 (23)	24 (18)
≥ 400	85 (19)	16 (17)	18 (21)	14 (11)
Dietary folate, μg, *n* (%)				
0–171	110 (25)	28 (31)	31 (37)	41 (31)
172–214	96 (22)	21 (23)	21 (25)	36 (27)
215–264	112 (26)	16 (17)	9 (11)	21 (16)
≥ 265	96 (22)	16 (17)	20 (24)	25 (19)
Missing	22 (5)	11 (12)	3 (3)	9 (7)
Multivitamins^d^, *n* (%)				
No	277 (64)	65 (71)	50 (60)	96 (73)
Yes	159 (36)	27 (29)	34 (40)	36 (27)
Education, *n* (%)				
Less than high school	45 (10)	20 (22)	9 (11)	24 (18)
High school and above	391 (90)	72 (78)	75 (89)	108 (82)

^a^During the first trimester

^b^Total number of drinks during the first trimester

^c^Any intake of folic acid supplements (either alone or with multivitamins)

^d^During month prior to pregnancy and first 2 months of pregnancy

### Orofacial cleft-associated CpG sites

We used unconditional logistic regression models to test whether the DNA methylation profile in blood of 2–3-day-old infants was associated with cleft phenotype at each CpG site on the array. Considering potential etiologic differences between cleft types, we first examined the association for CLO, CPO, and CLP separately, using as a reference the same group of unaffected control babies. After Bonferroni correction for multiple testing, there were no statistically significant associations for any of the analyses (Additional file 1: Figure S1), perhaps reflecting the small sample size in each case group. Consistent with other epidemiological studies, we then combined CLO and CLP case group as the “any cleft lip” group and repeated the association analysis (Additional file 1: Figure S2). This analysis identified one significant probe (cg09696939, *P* = 9.58 × 10^−8^) at gene BICC1 (Table 2). We also combined CPO with the CLP group as the “any cleft palate” group (Additional file 1: Figure S2) and identified another significant probe (cg26985354, *P* = 7.38 × 10^−8^) in CLASRP gene (Table 2). Additional file 2: Table S1 lists 43 CpG probes with *P* value less than 10^−5^ in any of the comparisons. Similar to Alvizi et al. [19], we compared controls with “any cleft” group (combining all three case groups together), but did not find any significant dmCpG at genome-wide significance level of 10^−7^. Similar to Sharp et al. [22], we also compared methylation profiles between cleft types (CLO vs CLP, CPO vs CLP, CLO vs CPO), but again did not identify any significant CpGs with *P* value < 10^−7^.

**Table 2:** CpGs associated with non-syndromic clefts

CpG	Chr	Position	Gene	Methylation	Coef.	*P*	Phenotype
cg09696939	10	60,272,079	BICC1	0.019	2.304	9.6 × 10^−8^	Any cleft lip
cg26985354	19	45,567,180	CLASRP	0.889	− 1.45	7.4 × 10^−8^	Any cleft palate

Epidemiological studies have suggested that multiple environmental exposures may affect the incidence of clefts. As a sensitivity analysis, we repeated the above association analyses while further adjusting for smoking, BMI, alcohol intake, folate intake, and gestational age. A total of 45 CpGs have association *P* values less than 10^−5^, and 30 of them overlap with the 43 CpGs from unadjusted analyses (Additional file 2: Table S2).

Using a Mendelian randomization analysis method with SNP and DNA methylation data in cord blood samples from the general population ALSPAC cohort, Howe et al. [21] identified 21 CpGs related to clefts. Four of the 21 CpGs did not pass quality control in our dataset. Additional file 2: Table S3 lists average methylation levels for the remaining 17 CpGs and related case-control association analysis results. We found statistically significant association for three of the CpGs at an unadjusted *P* value threshold of 0.05, including two (cg11398452 and cg02598441) of the five CpGs that passed a reverse-causation test reported by Howe et al. [21]. However, the direction of effect for two of the CpGs (cg02598441 and cg23166289) was reversed compared to Howe’s results. Although Howe’s and our NCL study show concordant direction of effect for CpG cg11398452, a Brazilian EWAS conducted by Alvizi et al. EWAS [19] reported an opposite direction of effect for this CpG. The Brazilian EWAS reported that 578 CpGs were differentially methylated between 59 controls and 67 combined cleft cases. Although 37 of these CpGs have unadjusted *P* value < 0.05 in our comparison of controls to combined cases, 33 (89%) have the opposite direction of effect (Additional file 2: Table S4).

Based on the GWAS Catalog (https://www.ebi.ac.uk/gwas/) and literature review of genetic linkage and association studies, we found 137 genes previously reported to be related to syndromic or non-syndromic clefts. Of the 3506 CpG probes on the Illumina 450K array located within 5 kb of these genes, 196 CpGs in 82 of these genes have association *P* value less than 0.01 (Additional file 2: Table S5). However, the proportion of small *P* value CpGs is not greater than array averages (binomial test *P* = 0.13), and no CpG in these genes had an association *P* value less than 10^−5^.

### Differentially methylated region analysis

We used two different methods to search for DMRs. Searches using DMRcate [30] software (with bandwidth at the default setting of 1000 nucleotides) found no significant DMRs. However, searches using Comb-P software identified a number of DMRs. In the comparisons of controls to individual cleft subtypes, we identified 10 DMRs for CLO, 6 for CPO, and 41 for CLP (Table 3, Additional file 2: Table S6). DMR (Chr6:28,583,971–28,584,289), comprising 14 CpGs in an intergenic region (between gene SCAND3 and LOC401242), was found in all three comparisons. Comparisons of controls to combined case subtypes identified 26 DMRs for CLP+CLO, 31 for CLP+CPO, and 37 DMRs when all case subtypes were combined. Some DMRs are found in multiple comparisons (Table 3, Additional file 2: Table S6). Similar to Sharp et al. [22], we also conducted DMR analysis between case subtypes and identified 10 DMRs in CLO compared to CPO, 9 DMRs in CLP compared to CLO, and 13 DMRs in CLP compared to CPO (Additional file 2: Table S6). DMR (17:80,541,737–80,542,007 in gene FOXK2) was identified in three comparisons (CLP vs CPO, CLO vs CPO, and control vs CPO) (Additional file 2: Table S6) and is also reported by Sharp’s study in one comparison (CLO vs CPO). EWAS results for individual CpGs within significant DMRs are listed in Additional file 2: Table S7.

**Table 3:** Number of significant DMRs and overlaps between them. The number of overlapped CpGs within each DMR is shown in parenthesis

EWAS	Case-control comparison						Case-case comparison		
	CLO	CPO	CLP	CLP+CLO	CLP+CPO	All case	CLO vs CPO	CLP vs CLO	CLP vs CPO
CLO	10 (75)	1 (14)	1 (14)	4 (28)	1 (14)	3 (22)	0 (0)	2 (21)	0 (0)
CPO		6 (33)	1 (14)	1 (14)	3 (19)	3 (19)	2 (7)	0 (0)	1 (2)
CLP			41 (275)	8 (67)	9 (73)	9 (76)	0 (0)	2 (10)	0 (0)
CLP+CLO				26 (184)	9 (83)	19 (134)	0 (0)	0 (0)	1 (4)
CLP+CPO					31 (198)	17 (118)	0 (0)	2 (10)	0 (0)
All case						37 (222)	0 (0)	0 (0)	0 (0)
CLO vs CPO							10 (45)	0 (0)	4 (21)
CLP vs CLO								9 (108)	0 (0)
CLP vs CPO									13 (64)

### Epigenetic age and orofacial cleft

Biological age estimated using DNA methylation data (epigenetic age) was recently reported to be associated with all-cause mortality [33]. Age acceleration (difference between epigenetic age and chronological age) could be used to indicate development rate in newborns. The blood samples in our study were collected from all babies 2–3 days after delivery, and thus, the chronological age is essentially 0 year old. We found that methylation age estimated using the Horvath method [34] was in general younger (~ 0.4 years) than the chronological age for our study participants. The means and standard deviations of epigenetic ages are − 0.39 ± 0.10, − 0.40 ± 0.11, − 0.41 ± 0.09, and − 0.40 ± 0.10, respectively, for controls, CLO, CPO, and CLP. Epigenetic age estimates for any of the cleft groups are slightly younger than those of the controls, but none of the pairwise comparisons are statistically significant (Tukey honest significant differences test, *P* > 0.1).

## Discussion

We conducted an epigenome-wide association study investigating 2–3-day-old infant blood DNA methylation and isolated orofacial clefts. We did not find individual dmCpG when we analyzed each cleft type separately. When we combined the cleft palate only with the cleft lip and palate, we identified one significantly methylated probe cg09696939 near gene BICC1. Similarly, when we combined the cleft lip only with the cleft lip and palate, we identified another significant dmCpG cg26985354 in gene CLASRP. Based on EWAS *P* values for individual CpGs and their chromosome location, we identified multiple differentially methylated regions (DMRs) between controls and case subtypes, and in case-case analysis of different subtypes. We confirmed one of the DMRs (17:80,541,737–80,542,007 in gene FOXK2) previously reported in case-case analysis by Sharp et al. [22].

CpG cg09696939 is located on chromosome 10, 824 base pairs upstream of gene BICC1. It is located in a CpG shore region and hypomethylated (averaged methylation level is 0.02). BICC1 encodes an RNA-binding protein, acts as a negative regulator of Wnt signaling, and is active in regulating gene expression by modulating protein translation during embryonic development. The gene is reported to be associated with cystic and renal dysplasia [35], myopia [36], and Alzheimer disease [37].

CpG cg26985354 is located on chromosome 19, in an intron of the gene CLASRP (CLK4-associating serine/arginine-rich protein). It is in a CpG island region and is hypermethylated (averaged methylation level is 0.89). CLASRP is a protein-coding gene, which functions as an alternative splicing regulator and may regulate members of the CLK kinase family [38].

Although we did not find any differentially methylated genomic regions using the DMRcate method [30], we found multiple DMRs using the Comp-P method [30], perhaps reflecting increased sensitivity of Comp-P when effect sizes are small. Compared to controls, CLP had the most DMRs (41 DMRs), followed by CLO (10 DMRs) and CPO (6 DMRs); only one DMR, in an intragenic region on chromosome 6, was shared among the three case subtypes. The number of DMRs found in each subtype could reflect sample size and resulting power (*n* = 132 CLP samples, 92 CLO samples, and 84 CPO samples). Subtype-specific DMRs might arise because of etiologic heterogeneity [22], but we cannot dismiss the possibility that they arise by chance.

In a study of a general population cohort without clefts, Howe et al. [21] found that cleft-associated polymorphisms predicted differential methylation of 21 CpGs. Although we found weak statistical support for three of these CpGs (threshold *P* < 0.05), the directions of effect for two were opposite from Howe’s prediction [21] and the third had an opposite direction of effect in an independent EWAS analysis of clefts [19, 21]. A study of blood DNA from 5-year-old children by Alvizi et al. [19] compared 67 combined cleft cases to 59 controls and reported 578 dmCpGs. But in our study, only 3 of these had the same direction of effect and met a threshold of unadjusted *P* < 0.05—fewer than expected by chance. A case-only study by Sharp et al. [22] of both blood and cleft tissue (with 50 samples of each cleft subtype) reported 335 dmCpGs when comparing CLO to CPO. However, we did not find any significant CpGs when performing similar case-case analysis in our NCL dataset. Sharp et al. also reported 121 DMRs, and we confirm one of the DMRs they report in the gene FOXK2. But while they found this DMR in a comparison of CLO vs CPO, we found it in CLO vs CPO, CLP vs CPO, and control vs CPO. Similar to Sharp et al., we also found that methylation age estimates for CLP were slightly higher than those for CLO and CPO although in our analysis none of these differences were statistically significant. The low replicate rate could be due to small sample size, age, and study design differences.

Unlike genotype, which remains constant, epigenetic profiles may change over time and in response to exposure. One of the most important strengths of our study is that blood samples for cases and controls used in analysis were uniformly collected and stored soon after delivery as part of a standardized PKU screening program across all regions of Norway, thus minimizing case-control differences in age, collection, handling, and storage. The study includes nationwide enrollment of all newborns referred for cleft surgery in Norway, with controls selected by random sampling from among all live births in Norway during the same period.

Our study also has limitations. Although larger than prior cleft-methylation studies, our sample size is still relatively small, particularly when considering specific cleft subtypes. Orofacial clefts result from fusion failures in very early pregnancy. We compared DNA methylation profiles between cleft cases and controls from blood samples at birth, and those results may not be an accurate surrogate for methylation differences in the relevant target tissues during early fetal life. Methylation differences in the blood of newborns could be caused by cleft-related differences in genetic or environmental exposures, but could also be the consequence of other unmeasured factors or due to chance.

## Conclusions

Using newborn blood DNA, we identified two CpGs and multiple genomic regions that are differentially methylated between controls and cleft cases, and additional differences between cleft subtypes. These findings lend some support to the hypothesis that aberrant DNA methylation profiles may be related to orofacial clefts.

## Additional files

Additional file 1: **Figure S1.** EWAS test results for comparisons between shared controls and cleft subtypes (CLO, CPO and CLP). Shown are the -log10(P) multiplied by sign of methylation coefficient estimated using unconditional logistic regression.** Figure S2.** EWAS test results for comparisons between shared controls and combined cleft subtypes (CLP+CPO and CLP+CLO). Shown are the -log10(P) multiplied by sign of methylation coefficient estimated using unconditional logistic regression. (DOCX 463 kb)

Additional file 2 **Table S1.** CpG probes with association *P* value less than 10^−5^ in any one of the cleft subtype analysis. Unconditional logistic regression model was used to test for association between DNA methylation M value and cleft status (shared controls vs. each cleft type). Covariables adjusted in each test are control surrogate variables, plate, gender, calendar year of baby’s birth, gestational age, and blood cell subtype composition. **Table S2.** Sensitivity analysis results by further adjusting for mother’s smoking status, BMI, drinking, folate intake and gestational age. Shown are CpG probes with association *P* value less than 10^−5^ in any one of the cleft subtype analysis. Unconditional logistic regression model was used to test for association between DNA methylation M value and cleft status (shared controls vs. each cleft type). Total covariables adjusted in each test are control surrogate variables, plate, gender, calendar year of baby’s birth, gestational age, blood cell subtype composition, smoking, BMI, drinking, folate intake and gestational age. **Table S3.** CpGs reported to be associated with cleft using Mendelian randomization analysis by Howe et al. 2018. Shown are the analysis results in NCL dataset: mean methylation level in control groups, all cleft case combined group(case), and case subgroups; case-control logistic regression estimated coefficient and *P* values for the combined case group and case subgroups. **Table S4.** Methylation means and association *P* values (controls compared to combined cleft cases) in Alvizi et al. (Scientific Reports, 2016) study and NCL study. Shown are the overlapped CpGs between NCL study and 578 CpGs reported by Alvizi et al. **Table S5.** Candidate gene results. Shown are CpGs around literature reported cleft-associated genes with methylation association *P* value less than 0.01 in any one of the cleft subtype analysis. **Table S6.** Differentially methylated regions (DMR) with Sidak multiple testing corrected *P* value < 0.05. **Table S7.** CpGs and association results within differentially methylated regions. (ZIP 229 kb)

## Abbreviations

ALSPAC: Avon Longitudinal Study of Parents and Children
BMI: Body mass index
CLO: Cleft lip only
CLP: Cleft lip and palate
CpG: Cytosine-guanine dinucleotide
CPO: Cleft palate only
dmCpG: Differentially methylated CpG
DMR: Differentially methylated region
ENmix: Exponential-Normal mixture signal intensity background correction
EWAS: Epigenome-wide association studies
GWAS: Genome-wide association studies
NCL: Norway Facial Clefts Study
PKU: Phenylketonuria
RCP: Regression on Correlated Probes
RELIC: REgression on Logarithm of Internal Control probes
UTR: Untranslated region
