# Supplementary material for: A comparison of DNA methylation in newborn blood samples from infants with and without orofacial clefts
Source: Clin Epigenetics. 2019 Mar 4;11:40. doi: 10.1186/s13148-019-0638-9 (PMC6399917; doi:10.1186/s13148-019-0638-9)

**Figure S1** EWAS test results for comparisons between shared controls and cleft subtypes (CLO,CPO and CLP). Shown are the -log10(P) multiplied by sign of methylation coefficient estimated using unconditional logistic regression.


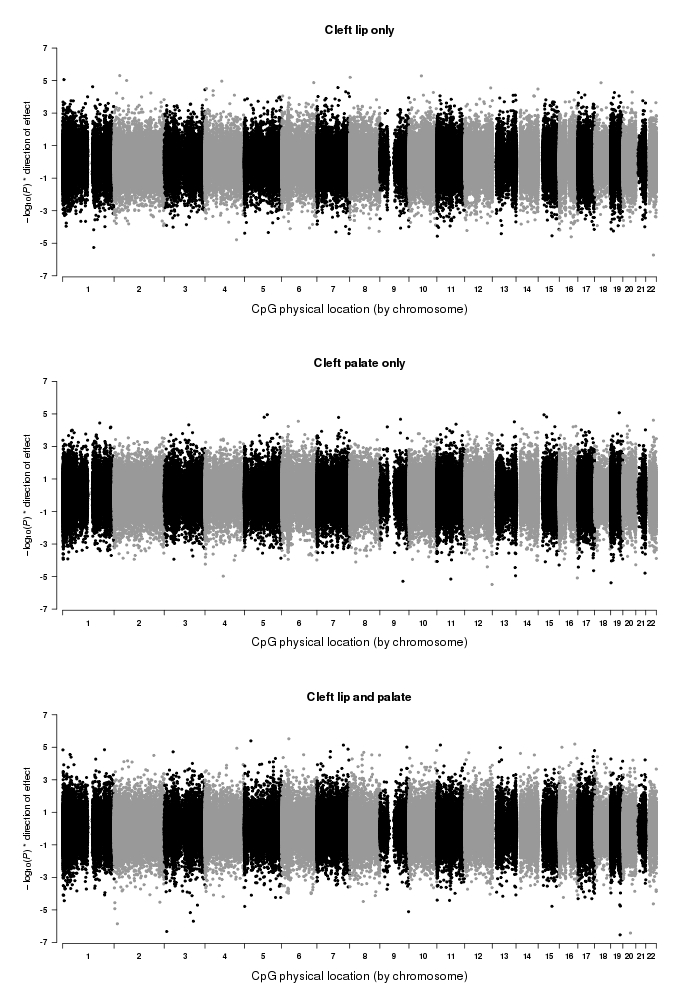


**Figure S2** EWAS test results for comparisons between shared controls and combined cleft subtypes (CLP+CPO and CLP+CLO). Shown are the -log10(P) multiplied by sign of methylation coefficient estimated using unconditional logistic regression.


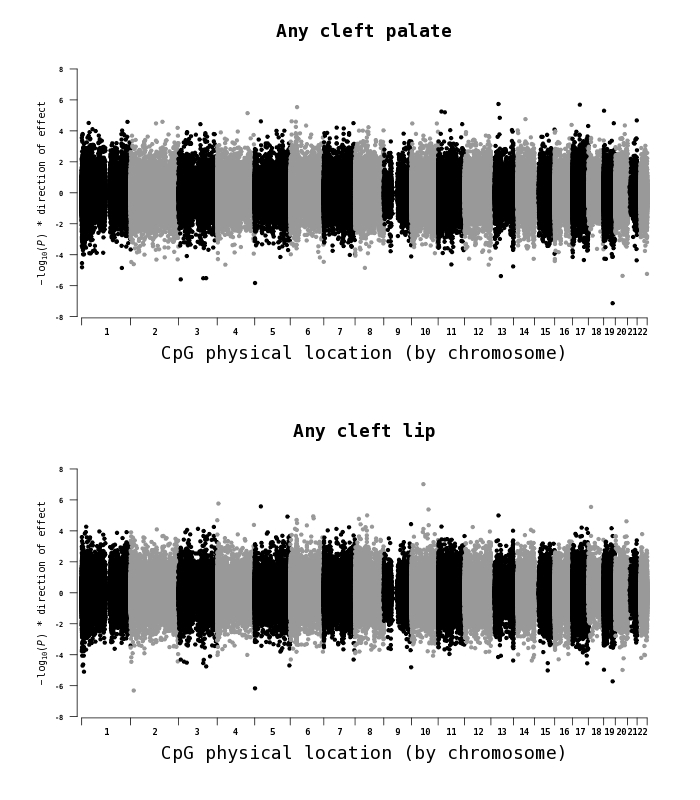

Supplement: Supplementary file 1 — Figure S1. EWAS test results for comparisons between shared controls and cleft subtypes (CLO, CPO and CLP). Shown are the -log10(P) multiplied by sign of methylation coefficient estimated using unconditional logistic regression. Figure S2. EWAS test results for comparisons between shared controls and combined cleft subtypes (CLP+CPO and CLP+CLO). Shown are the -log10(P) multiplied by sign of methylation coefficient estimated using unconditional logistic regression. (DOCX 463 kb) [file 13148_2019_638_MOESM1_ESM.docx]
