# Supplementary material for: A comparison of DNA methylation in newborn blood samples from infants with and without orofacial clefts
Source: Clin Epigenetics. 2019 Mar 4;11:40. doi: 10.1186/s13148-019-0638-9 (PMC6399917; doi:10.1186/s13148-019-0638-9)
Supplement: Supplementary file 2 — Table S1. CpG probes with association P value less than 10−5 in any one of the cleft subtype analysis. Unconditional logistic regression model was used to test for association between DNA methylation M value and cleft status (shared controls vs. each cleft type). Covariables adjusted in each test are control surrogate variables, plate, gender, calendar year of baby’s birth, gestational age, and blood cell subtype composition. Table S2. Sensitivity analysis results by further adjusting for mother’s smoking status, BMI, drinking, folate intake and gestational age. Shown are CpG probes with association P value less than 10−5 in any one of the cleft subtype analysis. Unconditional logistic regression model was used to test for association between DNA methylation M value and cleft status (shared controls vs. each cleft type). Total covariables adjusted in each test are control surrogate variables, plate, gender, calendar year of baby’s birth, gestational age, blood cell subtype composition, smoking, BMI, drinking, folate intake and gestational age. Table S3. CpGs reported to be associated with cleft using Mendelian randomization analysis by Howe et al. 2018. Shown are the analysis results in NCL dataset: mean methylation level in control groups, all cleft case combined group(case), and case subgroups; case-control logistic regression estimated coefficient and P values for the combined case group and case subgroups. Table S4. Methylation means and association P values (controls compared to combined cleft cases) in Alvizi et al. (Scientific Reports, 2016) study and NCL study. Shown are the overlapped CpGs between NCL study and 578 CpGs reported by Alvizi et al. Table S5. Candidate gene results. Shown are CpGs around literature reported cleft-associated genes with methylation association P value less than 0.01 in any one of the cleft subtype analysis. Table S6. Differentially methylated regions (DMR) with Sidak multiple testing corrected P value < 0.05. Table S7. CpGs and a [file 13148_2019_638_MOESM2_ESM.zip › suppTable.docx]

**Table S1.** CpG probes with association P value less than 10^-5^ in any one of the cleft sub-type analysis. Unconditional logistic regression model was used to test for association between DNA methylation M value and cleft status (shared controls vs. each cleft type). Covariables adjusted in each test are: control surrogate variables, plate, gender, calendar year of baby’s birth, gestational age, and blood cell subtype composition.

| **CpG** | **Chr** | **Position** | **Gene** | **CLO** | |  | **CPO** | |  | **CLP** | |  | **CLP+CPO** | |  | **CLP+CLO** | |
| --- | --- | --- | --- | --- | --- | --- | --- | --- | --- | --- | --- | --- | --- | --- | --- | --- | --- |
|  |  |  |  | **Coef.** | **P** |  | **Coef.** | **P** |  | **Coef.** | **P** |  | **Coef.** | **P** |  | **Coef.** | **P** |
| cg05927645 | 1 | 5943175 | NPHP4 | 2.86 | 8.7E-06 |  | 0.95 | 1.5E-01 |  | 0.24 | 6.4E-01 |  | 0.44 | 3.0E-01 |  | 1.08 | 9.8E-03 |
| cg04872402 | 1 | 11705983 |  | -2.05 | 6.8E-04 |  | -1.66 | 7.9E-03 |  | -1.85 | 2.0E-04 |  | -1.62 | 1.1E-04 |  | -1.86 | 8.0E-06 |
| cg07314391 | 1 | 150602100 | ENSA | -2.39 | 5.5E-06 |  | -0.75 | 1.8E-01 |  | -0.54 | 2.1E-01 |  | -0.68 | 6.4E-02 |  | -1.18 | 8.8E-04 |
| cg13914708 | 2 | 15804963 |  | -1.96 | 9.2E-04 |  | -0.51 | 4.5E-01 |  | -2.66 | 1.4E-06 |  | -1.90 | 2.5E-05 |  | -2.19 | 4.9E-07 |
| cg16212114 | 2 | 27193496 | MAPRE3 | 3.49 | 4.9E-06 |  | -0.14 | 8.6E-01 |  | -0.13 | 8.3E-01 |  | -0.04 | 9.5E-01 |  | 1.19 | 2.2E-02 |
| cg21898392 | 2 | 60983440 | PAPOLG | 2.54 | 9.9E-06 |  | 0.36 | 5.3E-01 |  | 0.53 | 2.3E-01 |  | 0.43 | 2.4E-01 |  | 1.17 | 1.4E-03 |
| cg25423174 | 3 | 12236303 |  | -1.09 | 1.4E-01 |  | -1.58 | 8.4E-02 |  | -3.98 | 4.7E-07 |  | -2.97 | 2.6E-06 |  | -2.36 | 4.8E-05 |
| cg18670236 | 3 | 126630478 | CHCHD6 | -0.96 | 2.9E-02 |  | -1.40 | 2.6E-03 |  | -1.63 | 6.8E-06 |  | -1.40 | 3.0E-06 |  | -1.23 | 2.9E-05 |
| cg15550350 | 3 | 141497168 | GRK7 | -1.02 | 2.0E-01 |  | -2.01 | 2.7E-02 |  | -3.20 | 2.0E-06 |  | -2.63 | 3.1E-06 |  | -2.27 | 1.8E-05 |
| cg17410394 | 4 | 5897891 | CRMP1 | 1.65 | 1.5E-04 |  | 0.16 | 7.3E-01 |  | 1.36 | 2.8E-04 |  | 0.89 | 3.3E-03 |  | 1.44 | 1.7E-06 |
| cg12614529 | 4 | 154269418 | MND1 | -0.30 | 7.4E-01 |  | 2.00 | 3.8E-02 |  | 3.44 | 1.1E-05 |  | 2.91 | 7.1E-06 |  | 1.85 | 2.8E-03 |
| cg26002008 | 5 | 1477719 | LPCAT1 | -3.68 | 2.6E-03 |  | -3.74 | 2.9E-03 |  | -4.53 | 1.6E-05 |  | -4.15 | 1.5E-06 |  | -4.31 | 6.7E-07 |
| cg21491163 | 5 | 31323216 | CDH6 | 1.69 | 1.9E-02 |  | 1.48 | 6.6E-02 |  | 3.03 | 4.1E-06 |  | 2.24 | 2.4E-05 |  | 2.43 | 2.6E-06 |
| cg04690793 | 6 | 34723390 | SNRPC | 0.71 | 2.1E-01 |  | 1.30 | 3.7E-02 |  | 2.26 | 3.0E-06 |  | 1.90 | 2.9E-06 |  | 1.63 | 3.1E-05 |
| cg07611843 | 7 | 128453143 | CCDC136 | 1.62 | 1.2E-01 |  | 0.70 | 5.5E-01 |  | 4.12 | 7.4E-06 |  | 2.95 | 1.4E-04 |  | 2.99 | 5.8E-05 |
| cg12284098 | 8 | 1993893 | MYOM2 | 2.37 | 6.3E-06 |  | 0.73 | 1.7E-01 |  | 0.37 | 4.3E-01 |  | 0.53 | 1.6E-01 |  | 1.25 | 1.2E-03 |
| cg20091636 | 8 | 61326482 | LOC100505532 | 2.11 | 4.1E-04 |  | 1.59 | 1.3E-02 |  | 1.65 | 8.0E-04 |  | 1.59 | 1.1E-04 |  | 1.79 | 1.0E-05 |
| cg25018881 | 9 | 111697645 | FAM206A | -1.30 | 1.0E-01 |  | -4.35 | 5.1E-06 |  | 0.33 | 6.3E-01 |  | -1.18 | 4.0E-02 |  | -0.34 | 5.4E-01 |
| cg07134045 | 9 | 130690198 | PIP5KL1 | -0.54 | 3.6E-01 |  | 0.16 | 7.9E-01 |  | 2.19 | 9.8E-06 |  | 1.40 | 4.7E-04 |  | 0.99 | 1.3E-02 |
| cg02516189 | 9 | 139268092 | CARD9 | -1.24 | 3.5E-02 |  | -0.87 | 2.1E-01 |  | -2.29 | 7.7E-06 |  | -1.72 | 7.7E-05 |  | -1.78 | 1.5E-05 |
| cg09696939 | 10 | 60272079 | BICC1 | 2.83 | 5.1E-06 |  | 1.04 | 8.7E-02 |  | 2.04 | 1.0E-04 |  | 1.58 | 2.0E-04 |  | 2.30 | 9.6E-08 |
| cg16223358 | 10 | 86372858 |  | 2.42 | 1.3E-03 |  | 0.90 | 2.6E-01 |  | 2.46 | 1.7E-04 |  | 1.72 | 1.3E-03 |  | 2.46 | 4.2E-06 |
| cg09072230 | 11 | 16918440 | PLEKHA7 | 0.98 | 1.4E-01 |  | 1.68 | 2.2E-02 |  | 2.57 | 7.3E-06 |  | 2.19 | 5.6E-06 |  | 1.88 | 5.3E-05 |
| cg23845009 | 11 | 34323678 | ABTB2 | 0.70 | 2.5E-01 |  | 2.05 | 2.8E-03 |  | 2.12 | 1.4E-04 |  | 2.10 | 6.3E-06 |  | 1.57 | 3.7E-04 |
| cg18192917 | 11 | 67401533 | NUDT8 | -0.08 | 9.2E-01 |  | -4.80 | 7.0E-06 |  | -0.21 | 7.7E-01 |  | -1.71 | 4.0E-03 |  | -0.24 | 6.7E-01 |
| cg21416739 | 12 | 132429311 |  | -2.57 | 3.4E-02 |  | -6.65 | 3.3E-06 |  | -2.29 | 2.9E-02 |  | -3.56 | 5.5E-05 |  | -2.35 | 5.4E-03 |
| cg17071063 | 13 | 38444679 | TRPC4 | 0.94 | 4.4E-02 |  | 1.38 | 6.6E-03 |  | 1.91 | 1.1E-05 |  | 1.68 | 1.8E-06 |  | 1.52 | 1.0E-05 |
| cg04949225 | 13 | 50796845 |  | -2.13 | 7.1E-02 |  | -4.05 | 2.0E-03 |  | -4.14 | 6.9E-05 |  | -3.93 | 4.2E-06 |  | -3.26 | 8.6E-05 |
| cg18091264 | 15 | 66795595 | SNORD18A | -2.17 | 1.2E-02 |  | 0.72 | 4.6E-01 |  | -3.33 | 1.7E-05 |  | -1.74 | 5.1E-03 |  | -2.71 | 9.6E-06 |
| cg00088691 | 16 | 12620513 | SNX29 | 0.06 | 9.5E-01 |  | -1.56 | 9.7E-02 |  | 3.34 | 1.0E-05 |  | 1.43 | 1.8E-02 |  | 1.80 | 2.8E-03 |
| cg03239970 | 16 | 75088935 | ZNRF1 | 0.24 | 6.6E-01 |  | 0.36 | 5.2E-01 |  | 2.35 | 6.4E-06 |  | 1.44 | 3.1E-04 |  | 1.43 | 5.2E-04 |
| cg04316925 | 16 | 87471211 | ZCCHC14 | 0.07 | 9.3E-01 |  | -3.94 | 8.2E-06 |  | -0.71 | 2.8E-01 |  | -1.79 | 1.3E-03 |  | -0.49 | 3.8E-01 |
| cg03572260 | 17 | 37808339 | STARD3 | 0.83 | 1.7E-01 |  | 2.26 | 3.7E-04 |  | 1.94 | 3.2E-04 |  | 2.08 | 2.1E-06 |  | 1.45 | 1.1E-03 |
| cg22454011 | 18 | 13611439 | LDLRAD4 | 1.94 | 4.1E-04 |  | 0.97 | 1.1E-01 |  | 1.93 | 9.5E-05 |  | 1.47 | 3.3E-04 |  | 1.91 | 2.8E-06 |
| cg10095020 | 19 | 1513078 | ADAMTSL5 | 0.10 | 8.4E-01 |  | -2.98 | 4.1E-06 |  | -0.25 | 5.9E-01 |  | -1.26 | 1.1E-03 |  | -0.09 | 7.9E-01 |
| cg08287334 | 19 | 1854633 | KLF16 | 0.03 | 9.5E-01 |  | 2.35 | 1.5E-03 |  | 1.87 | 5.3E-04 |  | 2.11 | 5.0E-06 |  | 1.01 | 1.5E-02 |
| cg27141871 | 19 | 41318235 |  | -0.22 | 7.4E-01 |  | 3.42 | 8.5E-06 |  | 0.92 | 1.1E-01 |  | 1.69 | 4.4E-04 |  | 0.44 | 3.4E-01 |
| cg26985354 | 19 | 45567180 | CLASRP | -0.66 | 5.6E-02 |  | -0.98 | 1.7E-02 |  | -1.63 | 2.9E-07 |  | -1.45 | 7.4E-08 |  | -1.20 | 1.9E-06 |
| cg16959758 | 20 | 36796513 | TGM2 | -2.25 | 1.2E-01 |  | -2.50 | 8.2E-02 |  | -6.26 | 3.8E-07 |  | -4.43 | 4.3E-06 |  | -4.32 | 1.0E-05 |
| cg01584939 | 22 | 37254322 | NCF4 | -4.34 | 1.9E-06 |  | -1.66 | 6.8E-02 |  | -0.26 | 7.0E-01 |  | -0.60 | 3.1E-01 |  | -1.73 | 2.5E-03 |
| cg09349128 | 22 | 50327986 |  | -0.27 | 7.7E-01 |  | -3.02 | 5.7E-03 |  | -3.18 | 1.4E-04 |  | -3.18 | 5.8E-06 |  | -1.92 | 2.9E-03 |
| cg05091491 | X | 114423926 | RBMXL3 | -1.94 | 1.3E-04 |  | -0.43 | 4.6E-01 |  | -1.54 | 6.0E-04 |  | -0.96 | 1.1E-02 |  | -1.65 | 9.3E-06 |
| cg10477471 | X | 131263364 | FRMD7 | -1.03 | 1.4E-01 |  | -0.39 | 6.0E-01 |  | -2.82 | 3.3E-06 |  | -1.88 | 1.2E-04 |  | -1.91 | 6.0E-05 |

**Table S2.** Sensitivity analysis results by further adjusting for mother’s smoking status, BMI, drinking, folate intake and gestational age. Shown are CpG probes with association P value less than 10^-5^ in any one of the cleft sub-type analysis. Unconditional logistic regression model was used to test for association between DNA methylation M value and cleft status (shared controls vs. each cleft type). Total covariables adjusted in each test are: control surrogate variables, plate, gender, calendar year of baby’s birth, gestational age, blood cell subtype composition, smoking, BMI, drinking, folate intake and gestational age.

| **CpG** | **Chr** | **Position** | **Gene** | **CLO** | |  | **CPO** | |  | **CLP** | |  | **CLP+CPO** | |  | **CLP+CLO** | |
| --- | --- | --- | --- | --- | --- | --- | --- | --- | --- | --- | --- | --- | --- | --- | --- | --- | --- |
|  |  |  |  | **Coef.** | **P** |  | **Coef.** | **P** |  | **Coef.** | **P** |  | **Coef.** | **P** |  | **Coef.** | **P** |
| cg20801637 | 1 | 1795408 | GNB1 | -1.25 | 9.9E-02 |  | -3.55 | 9.9E-05 |  | -2.15 | 3.3E-03 |  | -2.80 | 7.1E-06 |  | -1.80 | 1.8E-03 |
| cg09269935 | 1 | 4541555 | NA | -1.48 | 2.4E-02 |  | -0.06 | 9.4E-01 |  | -2.50 | 4.0E-05 |  | -1.69 | 6.0E-04 |  | -2.15 | 8.2E-06 |
| cg17178218 | 1 | 7352249 | CAMTA1 | -1.37 | 4.4E-02 |  | -0.71 | 3.5E-01 |  | -2.60 | 1.1E-05 |  | -1.97 | 5.7E-05 |  | -2.13 | 9.7E-06 |
| cg04872402 | 1 | 11705983 | NA | -2.32 | 3.6E-04 |  | -1.72 | 6.3E-03 |  | -2.01 | 9.7E-05 |  | -1.69 | 6.7E-05 |  | -2.02 | 3.3E-06 |
| cg13787775 | 1 | 2.05E+08 | NA | -0.82 | 2.0E-01 |  | -1.87 | 4.4E-03 |  | -2.55 | 1.6E-05 |  | -2.17 | 4.3E-06 |  | -1.66 | 3.6E-04 |
| cg13914708 | 2 | 15804963 | NA | -1.97 | 1.5E-03 |  | -0.52 | 4.5E-01 |  | -2.92 | 4.6E-07 |  | -1.94 | 2.2E-05 |  | -2.33 | 2.6E-07 |
| cg25423174 | 3 | 12236303 | NA | -1.38 | 7.8E-02 |  | -1.56 | 9.2E-02 |  | -4.40 | 9.9E-08 |  | -3.15 | 1.1E-06 |  | -2.77 | 5.6E-06 |
| cg15550350 | 3 | 1.41E+08 | GRK7 | -0.94 | 2.5E-01 |  | -2.04 | 2.8E-02 |  | -3.29 | 1.9E-06 |  | -2.64 | 4.6E-06 |  | -2.29 | 2.3E-05 |
| cg07342783 | 3 | 1.61E+08 | NA | -1.77 | 1.7E-02 |  | 0.22 | 7.8E-01 |  | -2.88 | 5.3E-06 |  | -1.60 | 1.4E-03 |  | -2.24 | 1.7E-05 |
| cg02694037 | 4 | 41125894 | APBB2 | -0.15 | 8.4E-01 |  | -1.98 | 1.7E-02 |  | -2.92 | 3.0E-05 |  | -2.60 | 6.8E-06 |  | -1.78 | 1.2E-03 |
| cg22608848 | 4 | 88258112 | HSD17B11 | -0.32 | 6.4E-01 |  | -3.56 | 8.2E-06 |  | -1.11 | 7.3E-02 |  | -1.95 | 1.2E-04 |  | -0.73 | 1.3E-01 |
| cg12614529 | 4 | 1.54E+08 | MND1 | -0.81 | 3.9E-01 |  | 1.93 | 4.7E-02 |  | 3.75 | 5.3E-06 |  | 2.97 | 6.8E-06 |  | 1.86 | 3.7E-03 |
| cg07380026 | 5 | 1296007 | TERT | -3.78 | 8.4E-06 |  | -0.60 | 5.1E-01 |  | -0.40 | 5.4E-01 |  | -0.37 | 5.1E-01 |  | -1.57 | 3.6E-03 |
| cg26002008 | 5 | 1477719 | LPCAT1 | -3.95 | 2.2E-03 |  | -3.51 | 5.2E-03 |  | -4.91 | 8.7E-06 |  | -4.16 | 2.3E-06 |  | -4.73 | 2.2E-07 |
| cg21491163 | 5 | 31323216 | CDH6 | 1.73 | 2.0E-02 |  | 1.41 | 8.2E-02 |  | 3.03 | 7.9E-06 |  | 2.17 | 5.9E-05 |  | 2.41 | 5.9E-06 |
| cg04690793 | 6 | 34723390 | SNRPC | 0.42 | 4.8E-01 |  | 1.16 | 6.6E-02 |  | 2.35 | 2.5E-06 |  | 1.87 | 6.0E-06 |  | 1.58 | 8.1E-05 |
| cg00037441 | 6 | 1.17E+08 | RFX6 | 2.94 | 3.3E-04 |  | 1.59 | 6.9E-02 |  | 2.37 | 1.4E-03 |  | 1.77 | 3.3E-03 |  | 2.74 | 6.4E-06 |
| cg12284098 | 8 | 1993893 | MYOM2 | 2.49 | 5.0E-06 |  | 0.80 | 1.5E-01 |  | 0.54 | 2.7E-01 |  | 0.60 | 1.2E-01 |  | 1.39 | 4.6E-04 |
| cg20091636 | 8 | 61326482 | LOC100505532 | 2.20 | 4.7E-04 |  | 1.56 | 1.6E-02 |  | 1.74 | 6.3E-04 |  | 1.62 | 1.1E-04 |  | 1.90 | 6.4E-06 |
| cg07134045 | 9 | 1.31E+08 | PIP5KL1 | -0.26 | 6.7E-01 |  | 0.22 | 7.3E-01 |  | 2.27 | 7.7E-06 |  | 1.48 | 2.9E-04 |  | 1.10 | 6.8E-03 |
| cg02516189 | 9 | 1.39E+08 | CARD9 | -1.23 | 4.5E-02 |  | -0.89 | 2.0E-01 |  | -2.39 | 5.6E-06 |  | -1.78 | 5.5E-05 |  | -1.85 | 1.5E-05 |
| cg09696939 | 10 | 60272079 | BICC1 | 2.89 | 1.3E-05 |  | 1.03 | 9.0E-02 |  | 1.97 | 2.2E-04 |  | 1.51 | 4.4E-04 |  | 2.27 | 3.3E-07 |
| cg09072230 | 11 | 16918440 | PLEKHA7 | 0.69 | 3.3E-01 |  | 1.62 | 3.0E-02 |  | 2.72 | 4.2E-06 |  | 2.20 | 6.7E-06 |  | 1.84 | 1.1E-04 |
| cg23845009 | 11 | 34323678 | ABTB2 | 0.45 | 4.8E-01 |  | 2.10 | 2.5E-03 |  | 2.17 | 1.8E-04 |  | 2.10 | 9.5E-06 |  | 1.51 | 9.1E-04 |
| cg18192917 | 11 | 67401533 | NUDT8 | -0.03 | 9.7E-01 |  | -5.01 | 4.8E-06 |  | -0.01 | 9.9E-01 |  | -1.66 | 5.8E-03 |  | -0.14 | 8.1E-01 |
| cg21416739 | 12 | 1.32E+08 | NA | -2.05 | 1.1E-01 |  | -6.78 | 3.4E-06 |  | -2.11 | 5.1E-02 |  | -3.46 | 1.1E-04 |  | -2.07 | 1.8E-02 |
| cg17071063 | 13 | 38444679 | TRPC4 | 0.92 | 5.5E-02 |  | 1.40 | 6.8E-03 |  | 2.10 | 3.2E-06 |  | 1.84 | 4.3E-07 |  | 1.66 | 3.9E-06 |
| cg23283614 | 14 | 60632532 | DHRS7 | 0.26 | 4.9E-01 |  | 1.34 | 6.4E-04 |  | 1.04 | 1.0E-03 |  | 1.18 | 9.6E-06 |  | 0.74 | 4.6E-03 |
| cg23513095 | 15 | 28272148 | OCA2 | 0.76 | 3.3E-01 |  | 4.64 | 4.9E-06 |  | 0.37 | 5.6E-01 |  | 1.64 | 1.9E-03 |  | 0.56 | 2.8E-01 |
| cg18091264 | 15 | 66795595 | SNORD18A | -2.22 | 1.4E-02 |  | 0.95 | 3.4E-01 |  | -3.56 | 7.3E-06 |  | -1.79 | 4.7E-03 |  | -2.91 | 4.2E-06 |
| cg00088691 | 16 | 12620513 | SNX29 | 0.14 | 8.8E-01 |  | -1.55 | 1.1E-01 |  | 3.75 | 3.2E-06 |  | 1.66 | 7.6E-03 |  | 2.02 | 1.3E-03 |
| cg03239970 | 16 | 75088935 | ZNRF1 | 0.19 | 7.5E-01 |  | 0.36 | 5.3E-01 |  | 2.51 | 3.7E-06 |  | 1.52 | 2.1E-04 |  | 1.50 | 4.5E-04 |
| cg04316925 | 16 | 87471211 | ZCCHC14 | 0.34 | 6.9E-01 |  | -4.07 | 7.4E-06 |  | -0.67 | 3.2E-01 |  | -1.77 | 1.7E-03 |  | -0.45 | 4.3E-01 |
| cg03572260 | 17 | 37808339 | STARD3 | 0.81 | 2.0E-01 |  | 2.30 | 3.4E-04 |  | 1.88 | 6.2E-04 |  | 2.01 | 5.5E-06 |  | 1.40 | 2.0E-03 |
| cg22454011 | 18 | 13611439 | LDLRAD4 | 1.96 | 7.0E-04 |  | 1.03 | 9.0E-02 |  | 1.95 | 1.3E-04 |  | 1.51 | 2.8E-04 |  | 1.89 | 6.7E-06 |
| cg10095020 | 19 | 1513078 | ADAMTSL5 | 0.26 | 5.9E-01 |  | -2.92 | 7.1E-06 |  | -0.25 | 6.0E-01 |  | -1.24 | 1.5E-03 |  | -0.02 | 9.5E-01 |
| cg27141871 | 19 | 41318235 | NA | -0.19 | 7.8E-01 |  | 3.67 | 4.1E-06 |  | 0.94 | 1.1E-01 |  | 1.73 | 4.0E-04 |  | 0.51 | 2.8E-01 |
| cg15742245 | 19 | 43857717 | CD177 | 0.10 | 8.2E-01 |  | -0.71 | 1.2E-01 |  | -1.68 | 5.5E-06 |  | -1.22 | 3.9E-05 |  | -0.89 | 2.2E-03 |
| cg26985354 | 19 | 45567180 | CLASRP | -0.61 | 9.3E-02 |  | -1.00 | 1.7E-02 |  | -1.76 | 1.1E-07 |  | -1.49 | 6.5E-08 |  | -1.27 | 1.3E-06 |
| cg16959758 | 20 | 36796513 | TGM2 | -1.97 | 2.0E-01 |  | -2.46 | 8.9E-02 |  | -6.63 | 2.2E-07 |  | -4.50 | 5.1E-06 |  | -4.41 | 1.4E-05 |
| cg23662138 | 21 | 46495607 | ADARB1 | 1.30 | 8.8E-03 |  | 2.43 | 5.5E-05 |  | 1.38 | 2.2E-03 |  | 1.68 | 7.5E-06 |  | 1.25 | 4.3E-04 |
| cg09051119 | 22 | 36721648 | MYH9 | -0.96 | 1.9E-01 |  | 0.09 | 9.2E-01 |  | -2.55 | 8.5E-06 |  | -1.68 | 2.7E-04 |  | -1.86 | 4.9E-05 |
| cg01584939 | 22 | 37254322 | NCF4 | -4.51 | 2.6E-06 |  | -1.61 | 8.6E-02 |  | -0.39 | 5.8E-01 |  | -0.57 | 3.3E-01 |  | -1.76 | 2.7E-03 |
| cg10477471 | X | 1.31E+08 | FRMD7 | -0.99 | 1.6E-01 |  | -0.45 | 5.4E-01 |  | -3.01 | 1.7E-06 |  | -2.04 | 5.2E-05 |  | -2.06 | 3.6E-05 |

**Table S3.** CpGs reported to be associated with cleft using Mendelian randomization analysis by Howe et al 2018. Shown are the analysis results in NCL dataset: mean methylation level in control groups, all cleft case combined group(case), and case sub-groups; Case control logistic regression estimated coefficient and P values for the combined case group and case sub-groups.

| **probe** | **gene** | **Mean Methylation Level** | | | | |  | **Coefficient** | | | |  | **P** | | | |
| --- | --- | --- | --- | --- | --- | --- | --- | --- | --- | --- | --- | --- | --- | --- | --- | --- |
|  |  | **Ctrl** | **Case** | **CLO** | **CPO** | **CLP** |  | **Case** | **CLO** | **CPO** | **CLP** |  | **Case** | **CLO** | **CPO** | **CLP** |
| cg26112574 |  | 0.962 | 0.964 | 0.966 | 0.963 | 0.963 |  | 0.223 | 0.582 | 0.51 | -0.08 |  | 0.351 | 0.128 | 0.215 | 0.802 |
| cg09549015 | F3 | 0.043 | 0.043 | 0.041 | 0.047 | 0.043 |  | -0.077 | -0.526 | 0.584 | -0.11 |  | 0.733 | 0.142 | 0.145 | 0.728 |
| cg23166289 | DIEXF | 0.125 | 0.133 | 0.121 | 0.14 | 0.137 |  | 0.078 | -0.291 | 0.589 | 0.068 |  | 0.57 | 0.171 | **0.022** | 0.716 |
| ch.8.2579072R |  | 0.008 | 0.008 | 0.007 | 0.008 | 0.008 |  | 0.032 | -0.31 | 0.721 | -0.05 |  | 0.897 | 0.419 | 0.086 | 0.892 |
| cg00750430 | SHTN1 | 0.297 | 0.298 | 0.301 | 0.304 | 0.292 |  | -0.032 | 0.098 | -0.177 | -0.18 |  | 0.902 | 0.806 | 0.693 | 0.609 |
| cg03968911 | SHTN1 | 0.751 | 0.751 | 0.756 | 0.745 | 0.751 |  | -0.371 | 0.404 | -0.288 | -0.87 |  | 0.291 | 0.465 | 0.628 | 0.07 |
| cg11398452 | VAX1 | 0.004 | 0.004 | 0.004 | 0.004 | 0.004 |  | -0.296 | 0.234 | -1.401 | -0 |  | 0.322 | 0.615 | **0.01** | 0.994 |
| cg04870120 |  | 0.583 | 0.581 | 0.606 | 0.596 | 0.554 |  | -0.168 | 0.597 | -0.09 | -0.89 |  | 0.452 | 0.091 | 0.821 | 0.005 |
| cg04194852 | TPM1 | 0.01 | 0.009 | 0.009 | 0.009 | 0.01 |  | -0.008 | -0.774 | -0.233 | 0.888 |  | 0.982 | 0.182 | 0.709 | 0.075 |
| cg18901140 |  | 0.461 | 0.458 | 0.449 | 0.47 | 0.456 |  | -0.279 | -0.911 | 0.013 | -0.11 |  | 0.379 | 0.078 | 0.981 | 0.801 |
| cg19788727 | NTN1 | 0.093 | 0.091 | 0.088 | 0.094 | 0.091 |  | 0.147 | -0.069 | 0.306 | 0.239 |  | 0.471 | 0.838 | 0.393 | 0.39 |
| cg02481697 | NTN1 | 0.392 | 0.395 | 0.39 | 0.412 | 0.388 |  | 0.046 | -0.17 | 0.473 | -0.06 |  | 0.827 | 0.609 | 0.214 | 0.833 |
| cg01862363 | NTN1 | 0.397 | 0.384 | 0.368 | 0.399 | 0.386 |  | -0.062 | -0.362 | 0.21 | -0.02 |  | 0.681 | 0.143 | 0.438 | 0.927 |
| cg16107528 | NTN1 | 0.872 | 0.869 | 0.865 | 0.885 | 0.863 |  | 0.007 | -0.235 | 0.416 | -0.05 |  | 0.968 | 0.386 | 0.164 | 0.827 |
| cg14501219 | LOC146880 | 0.567 | 0.573 | 0.581 | 0.573 | 0.566 |  | 0.426 | 1.285 | 0.048 | 0.06 |  | 0.331 | 0.069 | 0.948 | 0.921 |
| cg02598441 | LOC146880 | 0.047 | 0.043 | 0.041 | 0.044 | 0.043 |  | -0.511 | -0.893 | -0.634 | -0.386 |  | **0.025** | **0.017** | 0.13 | 0.217 |
| cg05254098 | MKNK2 | 0.018 | 0.017 | 0.018 | 0.017 | 0.017 |  | -0.56 | 0.163 | -1.48 | -0.87 |  | 0.23 | 0.82 | 0.069 | 0.179 |

**Table S4.** Methylation means and association P values (controls compared to combined cleft cases) in Alvizi et al (Scientific Reports, 2016) study and NCL study. Shown are the overlapped CpGs between NCL study and 578 CpGs reported by Alvizi et al.

**Table S5.** Candidate gene results. Shown are CpGs around literature reported cleft associated genes with methylation association P value less than 0.01 in any one of the cleft sub-type analysis.

| CpG | **Chr** | **Position** | **Gene** | **CLO** | |  | **CPO** | |  | **CLP** | |  | **CLP+CPO** | |  | **CLP+CLO** | |
| --- | --- | --- | --- | --- | --- | --- | --- | --- | --- | --- | --- | --- | --- | --- | --- | --- | --- |
|  |  |  |  | **Coef.** | **P** |  | **Coef.** | **P** |  | **Coef.** | **P** |  | **Coef.** | **P** |  | **Coef.** | **P** |
| cg23088157 | 1 | 11851316 | MTHFR | -1.21 | 4.0E-02 |  | -1.72 | 6.8E-03 |  | -0.74 | 1.3E-01 |  | -1.08 | 9.4E-03 |  | -0.96 | 1.9E-02 |
| cg10205548 | 1 | 18975719 | PAX7 | 0.70 | 1.2E-01 |  | 1.35 | 1.1E-02 |  | 0.91 | 2.1E-02 |  | 1.00 | 2.5E-03 |  | 0.81 | 1.1E-02 |
| cg09329407 | 1 | 19054906 | PAX7 | 2.90 | 8.8E-04 |  | -1.52 | 8.0E-02 |  | 0.18 | 8.0E-01 |  | -0.29 | 6.1E-01 |  | 1.26 | 2.9E-02 |
| cg22626525 | 1 | 20008900 | TMCO4 | 0.73 | 3.0E-01 |  | -2.45 | 4.4E-03 |  | 0.03 | 9.7E-01 |  | -0.68 | 2.2E-01 |  | 0.30 | 5.6E-01 |
| cg01722423 | 1 | 111150133 | KCNA2 | 2.15 | 1.3E-03 |  | 0.75 | 2.1E-01 |  | -0.81 | 9.7E-02 |  | -0.14 | 7.2E-01 |  | 0.21 | 6.0E-01 |
| cg26297344 | 1 | 111151163 | KCNA2 | 1.14 | 2.9E-02 |  | 2.07 | 9.4E-04 |  | 0.60 | 2.0E-01 |  | 1.02 | 9.3E-03 |  | 0.80 | 3.0E-02 |
| cg10074409 | 1 | 209979377 | IRF6 | -0.15 | 6.3E-01 |  | 0.97 | 5.9E-03 |  | -0.22 | 4.5E-01 |  | 0.17 | 4.6E-01 |  | -0.21 | 3.6E-01 |
| cg22442454 | 1 | 209979470 | IRF6 | -1.40 | 5.8E-02 |  | 1.04 | 2.2E-01 |  | -2.01 | 1.8E-03 |  | -0.90 | 8.7E-02 |  | -1.62 | 1.9E-03 |
| cg23283495 | 1 | 209979779 | IRF6 | 0.02 | 8.7E-01 |  | 0.59 | 1.1E-03 |  | -0.16 | 1.7E-01 |  | 0.05 | 6.1E-01 |  | -0.11 | 2.7E-01 |
| cg00903998 | 1 | 216897293 | ESRRG | 0.23 | 6.4E-01 |  | 1.62 | 6.6E-03 |  | 0.12 | 7.6E-01 |  | 0.57 | 1.1E-01 |  | 0.14 | 6.8E-01 |
| cg01537645 | 1 | 217110605 | ESRRG | -0.55 | 3.1E-01 |  | -2.11 | 4.8E-04 |  | -0.34 | 4.7E-01 |  | -0.83 | 3.1E-02 |  | -0.41 | 2.9E-01 |
| cg25851789 | 1 | 217309794 | ESRRG | 2.45 | 5.4E-04 |  | 1.66 | 2.8E-02 |  | 0.42 | 4.3E-01 |  | 0.84 | 7.5E-02 |  | 1.18 | 1.0E-02 |
| cg25755575 | 1 | 217311531 | ESRRG | -1.31 | 4.9E-03 |  | -1.49 | 6.9E-03 |  | -0.65 | 1.1E-01 |  | -0.83 | 1.7E-02 |  | -0.87 | 6.9E-03 |
| cg21646084 | 2 | 16802843 | FAM49A | -1.55 | 1.2E-01 |  | -3.29 | 3.5E-03 |  | -0.45 | 6.0E-01 |  | -1.27 | 7.4E-02 |  | -0.82 | 2.4E-01 |
| cg10106284 | 2 | 16847988 | FAM49A | 0.99 | 1.2E-02 |  | 0.56 | 1.9E-01 |  | 0.86 | 9.0E-03 |  | 0.64 | 2.0E-02 |  | 0.91 | 6.9E-04 |
| cg25002781 | 2 | 42166456 | C2orf91 | 0.50 | 4.5E-02 |  | -0.31 | 2.8E-01 |  | 0.50 | 2.5E-02 |  | 0.21 | 2.6E-01 |  | 0.48 | 7.1E-03 |
| cg22076676 | 2 | 43503935 | THADA | 3.29 | 2.1E-03 |  | -0.36 | 7.5E-01 |  | 1.18 | 1.8E-01 |  | 0.64 | 3.8E-01 |  | 1.89 | 1.1E-02 |
| cg10553672 | 2 | 43515279 | THADA | 0.25 | 7.7E-01 |  | 1.37 | 1.7E-01 |  | 2.16 | 8.8E-03 |  | 1.62 | 1.6E-02 |  | 1.34 | 3.8E-02 |
| cg16098064 | 2 | 43823287 | THADA | 0.47 | 2.9E-01 |  | 0.49 | 3.7E-01 |  | 0.98 | 1.6E-02 |  | 0.90 | 9.7E-03 |  | 0.75 | 2.1E-02 |
| cg03762820 | 2 | 43823654 | THADA | 0.06 | 8.5E-01 |  | -0.99 | 3.1E-03 |  | -0.34 | 1.7E-01 |  | -0.53 | 1.3E-02 |  | -0.19 | 3.5E-01 |
| cg06969469 | 2 | 121554817 | GLI2 | -2.37 | 2.2E-03 |  | -1.33 | 8.1E-02 |  | -0.21 | 7.2E-01 |  | -0.51 | 3.0E-01 |  | -0.98 | 5.5E-02 |
| cg07390459 | 2 | 121582002 | GLI2 | -0.87 | 4.2E-01 |  | -2.88 | 2.1E-02 |  | -2.98 | 3.2E-03 |  | -2.67 | 1.3E-03 |  | -1.91 | 1.6E-02 |
| cg02774705 | 2 | 121741143 | GLI2 | 0.21 | 6.8E-01 |  | 1.01 | 7.7E-02 |  | 1.04 | 2.5E-02 |  | 1.06 | 7.0E-03 |  | 0.69 | 6.7E-02 |
| cg01145396 | 2 | 233403063 | CHRNG | 2.47 | 9.3E-03 |  | 0.00 | 1.0E+00 |  | 2.10 | 1.4E-02 |  | 1.23 | 7.5E-02 |  | 2.43 | 4.8E-04 |
| cg11677744 | 2 | 235863589 | SH3BP4 | 0.44 | 4.6E-01 |  | 1.51 | 2.0E-02 |  | 0.92 | 6.7E-02 |  | 1.19 | 4.5E-03 |  | 0.70 | 8.5E-02 |
| cg23081527 | 2 | 235867063 | SH3BP4 | 0.71 | 2.4E-01 |  | 0.99 | 1.0E-01 |  | 1.70 | 1.8E-03 |  | 1.33 | 2.1E-03 |  | 1.37 | 1.9E-03 |
| cg08092258 | 2 | 235902219 | SH3BP4 | -0.73 | 2.7E-01 |  | -1.25 | 1.0E-01 |  | -1.62 | 7.3E-03 |  | -1.37 | 6.7E-03 |  | -1.20 | 1.2E-02 |
| cg25976672 | 2 | 235907796 | SH3BP4 | -0.02 | 9.8E-01 |  | 0.71 | 4.5E-01 |  | -2.16 | 6.4E-03 |  | -1.11 | 7.9E-02 |  | -1.07 | 8.6E-02 |
| cg06997259 | 2 | 235956326 | SH3BP4 | -0.40 | 5.5E-01 |  | -2.40 | 1.8E-03 |  | -0.09 | 8.8E-01 |  | -0.82 | 8.9E-02 |  | -0.21 | 6.5E-01 |
| cg03256955 | 3 | 30649017 | TGFBR2 | -1.70 | 1.8E-03 |  | -0.74 | 1.9E-01 |  | -0.21 | 6.5E-01 |  | -0.47 | 2.0E-01 |  | -0.83 | 2.4E-02 |
| cg15724876 | 3 | 30714671 | TGFBR2 | -1.46 | 4.8E-02 |  | -2.14 | 7.1E-03 |  | -0.34 | 5.8E-01 |  | -0.85 | 9.6E-02 |  | -0.79 | 1.3E-01 |
| cg05645292 | 3 | 59795625 | FHIT | 0.66 | 3.3E-01 |  | 1.77 | 1.5E-02 |  | 1.27 | 3.0E-02 |  | 1.37 | 5.8E-03 |  | 1.15 | 1.9E-02 |
| cg13745692 | 3 | 60065489 | FHIT | 1.38 | 8.3E-03 |  | 0.40 | 4.5E-01 |  | 0.92 | 3.4E-02 |  | 0.71 | 4.8E-02 |  | 1.12 | 2.3E-03 |
| cg17894779 | 3 | 60066405 | FHIT | 1.01 | 5.7E-02 |  | 0.12 | 8.2E-01 |  | 1.65 | 3.9E-04 |  | 1.08 | 4.3E-03 |  | 1.45 | 1.7E-04 |
| cg15931943 | 3 | 61236909 | FHIT | 2.12 | 3.5E-04 |  | 0.87 | 1.6E-01 |  | 0.07 | 8.7E-01 |  | 0.26 | 5.0E-01 |  | 0.84 | 2.6E-02 |
| cg19049316 | 3 | 61237063 | FHIT | -1.80 | 2.0E-03 |  | -1.09 | 6.8E-02 |  | -0.40 | 3.9E-01 |  | -0.65 | 9.5E-02 |  | -1.03 | 9.5E-03 |
| cg10131075 | 3 | 61237865 | FHIT | 0.39 | 3.8E-01 |  | 1.14 | 1.8E-02 |  | 0.74 | 5.9E-02 |  | 0.90 | 6.0E-03 |  | 0.59 | 6.1E-02 |
| cg01377807 | 3 | 99795240 | FILIP1L | 1.19 | 1.9E-02 |  | -1.15 | 3.8E-02 |  | 1.71 | 3.0E-04 |  | 0.59 | 1.2E-01 |  | 1.52 | 7.3E-05 |
| cg21723486 | 3 | 189349323 | TP63 | 0.88 | 3.1E-02 |  | 0.04 | 9.2E-01 |  | 0.87 | 6.3E-03 |  | 0.57 | 3.3E-02 |  | 0.80 | 2.6E-03 |
| cg09368199 | 3 | 189602720 | TP63 | 0.50 | 4.7E-01 |  | -2.76 | 6.8E-04 |  | -1.05 | 8.4E-02 |  | -1.57 | 2.0E-03 |  | -0.49 | 3.1E-01 |
| cg13207326 | 4 | 4860190 | MSX1 | 0.18 | 7.1E-01 |  | -1.08 | 6.4E-02 |  | -0.95 | 3.3E-02 |  | -1.01 | 7.7E-03 |  | -0.53 | 1.4E-01 |
| cg20588069 | 4 | 4860439 | MSX1 | -0.63 | 2.1E-01 |  | -1.53 | 8.3E-03 |  | -0.17 | 6.8E-01 |  | -0.55 | 1.2E-01 |  | -0.26 | 4.5E-01 |
| cg14167596 | 4 | 4862910 | MSX1 | -1.15 | 3.9E-02 |  | -1.03 | 1.0E-01 |  | -1.29 | 1.0E-02 |  | -1.11 | 8.0E-03 |  | -1.17 | 3.9E-03 |
| cg01785568 | 4 | 4864833 | MSX1 | 0.15 | 7.8E-01 |  | 0.23 | 6.9E-01 |  | -1.33 | 7.3E-03 |  | -0.67 | 9.7E-02 |  | -0.77 | 5.3E-02 |
| cg26789064 | 4 | 4866130 | MSX1 | -0.34 | 3.1E-01 |  | -0.39 | 2.7E-01 |  | -1.13 | 1.3E-04 |  | -0.80 | 9.0E-04 |  | -0.76 | 1.3E-03 |
| cg20311846 | 4 | 77356250 | SHROOM3 | 0.12 | 4.9E-01 |  | 0.59 | 1.6E-03 |  | 0.11 | 4.7E-01 |  | 0.32 | 1.1E-02 |  | 0.11 | 3.9E-01 |
| cg27417997 | 4 | 77356416 | SHROOM3 | -0.02 | 9.5E-01 |  | 0.83 | 4.3E-03 |  | -0.09 | 7.1E-01 |  | 0.31 | 1.0E-01 |  | -0.04 | 8.2E-01 |
| cg23843090 | 4 | 77504676 | SHROOM3 | 1.76 | 7.1E-03 |  | -0.09 | 8.9E-01 |  | 0.48 | 3.9E-01 |  | 0.24 | 6.0E-01 |  | 1.00 | 2.9E-02 |
| cg04503593 | 4 | 77579326 | SHROOM3 | -1.74 | 1.4E-02 |  | -0.41 | 5.8E-01 |  | -1.90 | 3.0E-03 |  | -1.35 | 8.3E-03 |  | -1.77 | 6.1E-04 |
| cg09258200 | 4 | 77669688 | SHROOM3 | 2.45 | 1.9E-03 |  | 0.32 | 6.9E-01 |  | 0.80 | 2.3E-01 |  | 0.50 | 3.6E-01 |  | 1.47 | 7.3E-03 |
| cg05919461 | 4 | 77688155 | SHROOM3 | -0.64 | 2.3E-01 |  | -1.58 | 9.8E-03 |  | -0.79 | 7.4E-02 |  | -1.09 | 4.7E-03 |  | -0.73 | 4.4E-02 |
| ch.4.1501247F | 4 | 77702631 | SHROOM3 | -0.07 | 9.1E-01 |  | 1.75 | 8.7E-03 |  | -0.63 | 2.0E-01 |  | 0.17 | 6.7E-01 |  | -0.25 | 5.3E-01 |
| cg18688704 | 4 | 157889520 | PDGFC | 1.84 | 6.9E-03 |  | -0.54 | 4.5E-01 |  | 0.35 | 5.2E-01 |  | 0.06 | 9.0E-01 |  | 0.84 | 6.2E-02 |
| cg12692069 | 4 | 157897045 | PDGFC | 0.22 | 6.7E-01 |  | 0.99 | 7.9E-02 |  | 1.03 | 2.2E-02 |  | 1.06 | 4.4E-03 |  | 0.66 | 7.5E-02 |
| cg17741014 | 5 | 80690220 | ACOT12 | 1.34 | 3.1E-03 |  | 1.34 | 8.7E-03 |  | 0.56 | 1.4E-01 |  | 0.87 | 8.2E-03 |  | 0.91 | 4.2E-03 |
| cg09825414 | 5 | 149736690 | TCOF1 | 0.44 | 5.9E-01 |  | -2.18 | 2.3E-02 |  | -1.60 | 3.5E-02 |  | -1.85 | 3.3E-03 |  | -0.76 | 2.0E-01 |
| cg13011928 | 6 | 10399281 | TFAP2A | -1.62 | 4.2E-03 |  | -1.02 | 9.7E-02 |  | -0.21 | 6.5E-01 |  | -0.38 | 3.4E-01 |  | -0.67 | 8.8E-02 |
| cg25472998 | 6 | 10413233 | TFAP2A | 0.21 | 6.9E-01 |  | 2.04 | 3.3E-03 |  | 0.06 | 8.9E-01 |  | 0.71 | 7.1E-02 |  | 0.13 | 7.4E-01 |
| cg03009363 | 6 | 10419400 | TFAP2A | -1.78 | 4.1E-04 |  | -0.01 | 9.9E-01 |  | -0.31 | 4.6E-01 |  | -0.24 | 5.2E-01 |  | -0.84 | 1.6E-02 |
| cg18732032 | 6 | 10419867 | TFAP2A | 2.54 | 7.5E-03 |  | 1.30 | 1.9E-01 |  | 1.67 | 5.0E-02 |  | 1.40 | 4.5E-02 |  | 2.13 | 1.7E-03 |
| cg07492757 | 6 | 10420035 | TFAP2A | -0.17 | 7.6E-01 |  | 0.98 | 1.1E-01 |  | 1.61 | 1.3E-03 |  | 1.35 | 1.0E-03 |  | 0.87 | 2.9E-02 |
| cg08421910 | 6 | 11771840 | ADTRP | 1.07 | 2.0E-02 |  | 1.30 | 1.8E-03 |  | 0.19 | 6.3E-01 |  | 0.57 | 6.0E-02 |  | 0.58 | 7.8E-02 |
| cg14178895 | 6 | 11778902 | ADTRP | 2.23 | 8.7E-03 |  | 0.55 | 5.1E-01 |  | 0.71 | 2.8E-01 |  | 0.48 | 3.8E-01 |  | 1.23 | 2.8E-02 |
| cg11210880 | 6 | 11779911 | ADTRP | 0.09 | 8.4E-01 |  | 0.08 | 8.7E-01 |  | 1.06 | 8.2E-03 |  | 0.57 | 8.7E-02 |  | 0.67 | 4.2E-02 |
| cg02890435 | 6 | 33130918 | COL11A2 | 0.83 | 2.4E-01 |  | 0.13 | 8.7E-01 |  | -2.34 | 8.0E-04 |  | -1.36 | 1.7E-02 |  | -0.73 | 1.6E-01 |
| cg10390980 | 6 | 33131862 | COL11A2 | -0.01 | 9.9E-01 |  | -2.47 | 6.4E-03 |  | 0.22 | 7.2E-01 |  | -0.53 | 3.2E-01 |  | 0.08 | 8.7E-01 |
| cg20506659 | 6 | 33132943 | COL11A2 | -0.42 | 4.4E-01 |  | 1.53 | 7.3E-03 |  | -0.11 | 8.1E-01 |  | 0.45 | 2.3E-01 |  | -0.20 | 5.9E-01 |
| cg01693662 | 6 | 33136023 | COL11A2 | 0.71 | 5.3E-01 |  | -3.90 | 3.1E-03 |  | -1.69 | 9.3E-02 |  | -2.19 | 8.4E-03 |  | -0.71 | 3.7E-01 |
| cg24039329 | 6 | 33136996 | COL11A2 | 2.04 | 1.1E-01 |  | -4.45 | 6.4E-03 |  | -0.08 | 9.4E-01 |  | -1.05 | 2.6E-01 |  | 0.60 | 4.9E-01 |
| cg05636843 | 6 | 33138669 | COL11A2 | 0.04 | 9.6E-01 |  | -2.25 | 5.9E-03 |  | -0.88 | 1.4E-01 |  | -1.18 | 1.9E-02 |  | -0.59 | 2.2E-01 |
| cg08241373 | 6 | 33139529 | COL11A2 | 1.21 | 6.9E-02 |  | 1.37 | 6.0E-02 |  | 1.15 | 4.7E-02 |  | 1.25 | 9.8E-03 |  | 1.13 | 1.6E-02 |
| cg26079695 | 6 | 33143273 | COL11A2 | -1.98 | 7.0E-03 |  | 0.36 | 6.6E-01 |  | -1.08 | 9.1E-02 |  | -0.58 | 2.8E-01 |  | -1.31 | 1.3E-02 |
| cg22615515 | 6 | 33144975 | COL11A2 | -2.02 | 2.1E-03 |  | -0.74 | 3.3E-01 |  | -0.43 | 4.3E-01 |  | -0.59 | 2.1E-01 |  | -1.02 | 2.4E-02 |
| cg09261794 | 6 | 33147897 | COL11A2 | -0.40 | 4.3E-01 |  | 0.74 | 2.2E-01 |  | 1.26 | 6.5E-03 |  | 1.02 | 1.0E-02 |  | 0.56 | 1.3E-01 |
| cg27512176 | 6 | 33158538 | COL11A2 | -0.83 | 1.3E-01 |  | 1.69 | 7.7E-03 |  | 0.34 | 5.0E-01 |  | 0.90 | 3.0E-02 |  | -0.14 | 7.2E-01 |
| cg12936423 | 6 | 33160849 | COL11A2 | 0.89 | 1.0E-01 |  | 0.24 | 6.6E-01 |  | 1.07 | 2.5E-02 |  | 0.68 | 7.3E-02 |  | 1.01 | 9.7E-03 |
| cg25459558 | 6 | 33161290 | COL11A2 | 0.22 | 6.3E-01 |  | -0.89 | 7.6E-02 |  | -0.99 | 1.7E-02 |  | -0.94 | 5.7E-03 |  | -0.41 | 2.2E-01 |
| cg26357820 | 6 | 33161849 | COL11A2 | -1.74 | 6.1E-03 |  | 0.55 | 4.2E-01 |  | 0.39 | 4.6E-01 |  | 0.42 | 3.4E-01 |  | -0.39 | 3.7E-01 |
| cg02915381 | 6 | 33163333 | COL11A2 | -1.65 | 2.8E-02 |  | -1.34 | 9.1E-02 |  | -1.10 | 9.8E-02 |  | -1.18 | 3.1E-02 |  | -1.39 | 9.7E-03 |
| cg06370855 | 6 | 33163347 | COL11A2 | -0.18 | 7.5E-01 |  | -1.69 | 7.3E-03 |  | -0.40 | 4.1E-01 |  | -0.82 | 4.1E-02 |  | -0.26 | 5.1E-01 |
| cg01741041 | 7 | 5567862 | ACTB | 0.50 | 5.4E-01 |  | 0.81 | 3.8E-01 |  | 2.04 | 7.5E-03 |  | 1.42 | 2.5E-02 |  | 1.29 | 3.7E-02 |
| cg13677897 | 7 | 5570593 | ACTB | 1.49 | 2.5E-03 |  | -0.48 | 3.7E-01 |  | 0.27 | 5.5E-01 |  | -0.13 | 7.3E-01 |  | 0.81 | 2.3E-02 |
| cg27334919 | 7 | 19158378 | TWIST1 | 0.54 | 2.3E-01 |  | 1.30 | 7.0E-03 |  | 0.87 | 2.4E-02 |  | 0.99 | 2.0E-03 |  | 0.77 | 1.5E-02 |
| cg02709068 | 7 | 42016844 | GLI3 | -0.51 | 2.6E-01 |  | -0.60 | 2.5E-01 |  | -0.97 | 1.6E-02 |  | -0.85 | 1.4E-02 |  | -0.88 | 8.7E-03 |
| cg01013023 | 7 | 42131124 | GLI3 | -1.89 | 9.5E-03 |  | 0.27 | 7.1E-01 |  | -0.88 | 1.4E-01 |  | -0.48 | 3.3E-01 |  | -1.27 | 1.2E-02 |
| cg21466107 | 7 | 42136966 | GLI3 | -2.25 | 4.0E-03 |  | 0.67 | 4.0E-01 |  | 0.07 | 9.2E-01 |  | 0.22 | 6.9E-01 |  | -0.79 | 1.4E-01 |
| cg09847820 | 7 | 42261049 | GLI3 | -2.49 | 4.2E-02 |  | -0.74 | 5.5E-01 |  | -2.33 | 2.8E-02 |  | -1.60 | 6.1E-02 |  | -2.40 | 4.9E-03 |
| cg10950272 | 7 | 42276881 | GLI3 | 2.28 | 3.2E-04 |  | -0.31 | 6.0E-01 |  | 0.29 | 5.5E-01 |  | 0.07 | 8.7E-01 |  | 1.00 | 1.7E-02 |
| cg27378814 | 8 | 12941186 | DLC1 | 0.50 | 1.9E-01 |  | 1.40 | 2.5E-03 |  | 0.77 | 2.9E-02 |  | 0.99 | 6.8E-04 |  | 0.72 | 1.1E-02 |
| cg27395342 | 8 | 13224310 | DLC1 | -2.05 | 1.6E-02 |  | 0.18 | 8.3E-01 |  | -2.02 | 6.8E-03 |  | -1.06 | 7.7E-02 |  | -1.80 | 3.3E-03 |
| cg24946597 | 8 | 31497464 | NRG1 | 0.38 | 4.9E-01 |  | 1.01 | 1.1E-01 |  | 1.40 | 6.3E-03 |  | 1.30 | 2.0E-03 |  | 0.95 | 1.7E-02 |
| cg25353930 | 8 | 32078254 | NRG1 | -0.64 | 2.3E-01 |  | -1.77 | 4.8E-03 |  | 0.56 | 2.2E-01 |  | -0.30 | 4.5E-01 |  | 0.10 | 7.9E-01 |
| cg05303690 | 8 | 32284962 | NRG1 | -2.60 | 9.6E-04 |  | -0.59 | 4.6E-01 |  | -0.35 | 5.9E-01 |  | -0.31 | 5.7E-01 |  | -1.31 | 1.6E-02 |
| cg00400221 | 8 | 38323291 | FGFR1 | -0.68 | 2.4E-01 |  | -1.51 | 2.0E-02 |  | -1.22 | 1.5E-02 |  | -1.38 | 1.2E-03 |  | -1.05 | 1.2E-02 |
| cg06806638 | 8 | 61596860 | CHD7 | -0.40 | 3.1E-01 |  | -0.20 | 6.7E-01 |  | -1.07 | 2.7E-03 |  | -0.69 | 1.8E-02 |  | -0.79 | 5.0E-03 |
| cg26441877 | 8 | 61626625 | CHD7 | -0.15 | 7.9E-01 |  | 1.65 | 1.2E-02 |  | 1.21 | 1.5E-02 |  | 1.39 | 1.2E-03 |  | 0.60 | 1.2E-01 |
| cg07154931 | 8 | 95487232 | RAD54B | -0.83 | 9.8E-02 |  | -0.08 | 8.8E-01 |  | -0.92 | 2.4E-02 |  | -0.54 | 1.2E-01 |  | -0.95 | 5.7E-03 |
| cg22736280 | 8 | 95565249 | KIAA1429 | 2.32 | 3.0E-03 |  | 0.82 | 2.7E-01 |  | -0.28 | 6.5E-01 |  | 0.18 | 7.2E-01 |  | 0.62 | 2.2E-01 |
| cg00163859 | 8 | 99917203 | STK3 | -2.43 | 3.2E-04 |  | -1.66 | 2.4E-02 |  | -1.13 | 4.4E-02 |  | -1.28 | 7.4E-03 |  | -1.55 | 7.1E-04 |
| cg06070421 | 8 | 100026087 | VPS13B | 0.31 | 6.2E-01 |  | 2.57 | 1.2E-03 |  | 0.12 | 8.1E-01 |  | 0.87 | 6.0E-02 |  | 0.01 | 9.9E-01 |
| cg00451651 | 8 | 100252466 | VPS13B | 0.29 | 6.0E-01 |  | 0.00 | 1.0E+00 |  | 1.36 | 6.5E-03 |  | 0.77 | 5.7E-02 |  | 0.88 | 2.8E-02 |
| cg03492094 | 9 | 91994786 | SEMA4D | -0.86 | 2.7E-01 |  | 0.54 | 5.4E-01 |  | 2.07 | 2.9E-03 |  | 1.58 | 6.4E-03 |  | 0.67 | 2.2E-01 |
| cg14541848 | 9 | 91995929 | SEMA4D | 0.62 | 3.7E-01 |  | -2.83 | 8.6E-04 |  | -0.79 | 2.1E-01 |  | -1.15 | 2.8E-02 |  | -0.07 | 8.8E-01 |
| cg14298589 | 9 | 98215821 | PTCH1 | 0.22 | 7.7E-01 |  | 0.14 | 8.6E-01 |  | -1.64 | 8.7E-03 |  | -1.11 | 3.4E-02 |  | -0.83 | 1.1E-01 |
| cg00638945 | 9 | 98266643 | PTCH1 | -0.75 | 6.9E-02 |  | -0.23 | 6.0E-01 |  | -0.92 | 1.2E-02 |  | -0.65 | 2.9E-02 |  | -0.85 | 4.3E-03 |
| cg13428213 | 9 | 98267529 | PTCH1 | -0.65 | 3.7E-02 |  | -0.10 | 7.5E-01 |  | -0.61 | 2.2E-02 |  | -0.42 | 5.0E-02 |  | -0.60 | 5.8E-03 |
| cg14319249 | 9 | 98269526 | PTCH1 | -1.51 | 3.8E-03 |  | -0.68 | 2.3E-01 |  | -0.45 | 3.3E-01 |  | -0.57 | 1.3E-01 |  | -0.80 | 3.1E-02 |
| cg13754720 | 9 | 98274853 | PTCH1 | -0.94 | 6.3E-02 |  | -0.15 | 7.7E-01 |  | -0.95 | 2.9E-02 |  | -0.68 | 5.7E-02 |  | -0.97 | 7.0E-03 |
| cg13564742 | 9 | 100614879 | FOXE1 | 0.71 | 1.0E-01 |  | 0.70 | 1.7E-01 |  | 1.17 | 4.4E-03 |  | 0.92 | 5.9E-03 |  | 0.97 | 2.4E-03 |
| cg17702692 | 9 | 100617370 | FOXE1 | 2.52 | 6.4E-04 |  | 0.70 | 3.1E-01 |  | 0.18 | 7.3E-01 |  | 0.42 | 3.5E-01 |  | 1.03 | 2.6E-02 |
| cg13443911 | 9 | 101866107 | TGFBR1 | -1.23 | 7.1E-02 |  | 0.34 | 6.4E-01 |  | -1.50 | 1.2E-02 |  | -0.74 | 1.3E-01 |  | -1.45 | 3.1E-03 |
| cg14426785 | 9 | 101876671 | TGFBR1 | 1.89 | 2.2E-03 |  | 1.70 | 1.8E-02 |  | 1.21 | 2.4E-02 |  | 1.26 | 5.6E-03 |  | 1.50 | 5.4E-04 |
| cg23781495 | 9 | 118917109 | PAPPA | -0.34 | 5.6E-01 |  | 1.97 | 8.2E-03 |  | 0.08 | 8.7E-01 |  | 0.70 | 1.1E-01 |  | -0.06 | 8.7E-01 |
| cg14377982 | 9 | 127533798 | NR6A1 | -0.33 | 5.8E-01 |  | -1.78 | 9.5E-03 |  | -0.48 | 3.4E-01 |  | -0.74 | 7.8E-02 |  | -0.45 | 2.6E-01 |
| cg01179268 | 10 | 99696049 | CRTAC1 | 3.15 | 2.2E-02 |  | -0.83 | 5.6E-01 |  | 1.99 | 1.0E-01 |  | 0.74 | 4.5E-01 |  | 2.55 | 9.3E-03 |
| cg19479373 | 10 | 99734154 | CRTAC1 | 0.06 | 8.3E-01 |  | 0.86 | 9.6E-03 |  | 0.49 | 5.9E-02 |  | 0.56 | 7.8E-03 |  | 0.26 | 2.0E-01 |
| cg23245905 | 10 | 99735133 | CRTAC1 | 0.02 | 9.6E-01 |  | 1.00 | 2.1E-02 |  | 0.84 | 1.2E-02 |  | 0.90 | 1.9E-03 |  | 0.41 | 1.3E-01 |
| cg08930881 | 10 | 99735202 | CRTAC1 | 0.61 | 5.9E-02 |  | 0.73 | 3.6E-02 |  | 0.87 | 2.0E-03 |  | 0.80 | 6.5E-04 |  | 0.69 | 2.5E-03 |
| cg12098963 | 10 | 99789825 | CRTAC1 | 3.14 | 2.2E-04 |  | 0.91 | 2.9E-01 |  | 0.83 | 2.4E-01 |  | 0.75 | 1.9E-01 |  | 1.63 | 4.7E-03 |
| cg16079364 | 10 | 103532798 | FGF8 | 0.74 | 1.2E-01 |  | 0.98 | 7.1E-02 |  | 1.11 | 1.6E-02 |  | 0.93 | 1.3E-02 |  | 0.91 | 9.8E-03 |
| cg11206312 | 10 | 103535398 | FGF8 | -2.56 | 4.2E-03 |  | -0.87 | 3.3E-01 |  | -1.58 | 3.7E-02 |  | -1.22 | 4.7E-02 |  | -1.87 | 2.4E-03 |
| cg27533288 | 10 | 118896776 | VAX1 | 0.47 | 4.2E-01 |  | -1.96 | 4.3E-03 |  | -0.64 | 1.8E-01 |  | -0.90 | 2.7E-02 |  | -0.25 | 5.3E-01 |
| cg03690956 | 10 | 118900324 | VAX1 | -1.12 | 2.2E-02 |  | -1.05 | 6.1E-02 |  | -0.96 | 2.6E-02 |  | -0.96 | 8.6E-03 |  | -0.96 | 6.9E-03 |
| cg23936294 | 10 | 123325021 | FGFR2 | -1.78 | 2.5E-03 |  | -0.56 | 3.5E-01 |  | -0.09 | 8.6E-01 |  | -0.25 | 5.3E-01 |  | -0.67 | 9.7E-02 |
| cg14856220 | 10 | 123353500 | FGFR2 | -1.90 | 2.7E-03 |  | 0.36 | 6.0E-01 |  | -0.50 | 3.8E-01 |  | -0.12 | 7.9E-01 |  | -0.95 | 3.5E-02 |
| cg10314760 | 10 | 123353654 | FGFR2 | -1.34 | 8.4E-02 |  | -0.65 | 4.3E-01 |  | -1.96 | 3.5E-03 |  | -1.27 | 2.1E-02 |  | -1.66 | 2.2E-03 |
| cg25588697 | 11 | 71149985 | DHCR7 | 2.45 | 3.7E-03 |  | 0.82 | 4.1E-01 |  | 0.23 | 7.6E-01 |  | 0.32 | 6.1E-01 |  | 1.08 | 7.4E-02 |
| cg01892327 | 11 | 71159316 | DHCR7 | -1.58 | 8.7E-03 |  | 0.16 | 8.0E-01 |  | -0.59 | 2.2E-01 |  | -0.33 | 4.0E-01 |  | -0.89 | 2.7E-02 |
| cg14630206 | 11 | 77121864 | PAK1 | 2.64 | 1.9E-03 |  | 1.10 | 2.2E-01 |  | 0.89 | 2.0E-01 |  | 0.88 | 1.2E-01 |  | 1.53 | 7.3E-03 |
| cg20232503 | 11 | 77125157 | PAK1 | 0.20 | 8.0E-01 |  | 2.47 | 3.2E-03 |  | -0.74 | 2.6E-01 |  | 0.47 | 3.8E-01 |  | -0.34 | 5.3E-01 |
| cg03213860 | 11 | 77185670 | PAK1 | 0.52 | 1.5E-01 |  | 1.19 | 2.9E-03 |  | 0.15 | 6.4E-01 |  | 0.53 | 4.7E-02 |  | 0.22 | 3.9E-01 |
| cg25461513 | 11 | 115096810 | CADM1 | -0.56 | 3.5E-01 |  | -1.69 | 9.6E-03 |  | -0.71 | 1.4E-01 |  | -0.92 | 2.3E-02 |  | -0.61 | 1.4E-01 |
| cg12848345 | 11 | 115373119 | CADM1 | 0.68 | 1.5E-01 |  | 0.44 | 3.7E-01 |  | 1.29 | 1.5E-03 |  | 0.94 | 6.1E-03 |  | 1.02 | 2.5E-03 |
| cg10193817 | 11 | 115375226 | CADM1 | 0.19 | 7.2E-01 |  | 1.73 | 9.6E-03 |  | 0.92 | 7.0E-02 |  | 1.04 | 1.3E-02 |  | 0.50 | 2.1E-01 |
| cg08066991 | 11 | 115376699 | CADM1 | 0.01 | 9.8E-01 |  | 1.57 | 7.0E-03 |  | 0.12 | 7.9E-01 |  | 0.64 | 9.1E-02 |  | 0.18 | 6.4E-01 |
| cg16067696 | 11 | 119522486 | PVRL1 | -1.28 | 7.2E-02 |  | -0.39 | 6.0E-01 |  | -1.67 | 3.9E-03 |  | -1.20 | 1.4E-02 |  | -1.46 | 2.6E-03 |
| cg26921987 | 11 | 119548369 | PVRL1 | -0.34 | 6.1E-01 |  | -2.11 | 4.1E-03 |  | -0.14 | 8.1E-01 |  | -0.81 | 8.6E-02 |  | -0.27 | 5.6E-01 |
| cg25577524 | 11 | 119560689 | PVRL1 | -0.56 | 4.3E-01 |  | -2.45 | 2.2E-03 |  | -1.42 | 2.1E-02 |  | -1.61 | 1.7E-03 |  | -1.02 | 3.8E-02 |
| cg26934062 | 11 | 125757419 | HYLS1 | 0.45 | 4.1E-01 |  | 1.27 | 4.7E-02 |  | 1.47 | 2.5E-03 |  | 1.30 | 1.6E-03 |  | 1.05 | 6.3E-03 |
| cg13479898 | 11 | 125757446 | HYLS1 | 1.55 | 8.3E-03 |  | 0.67 | 2.7E-01 |  | -0.85 | 7.7E-02 |  | -0.29 | 4.6E-01 |  | 0.05 | 8.9E-01 |
| cg20255775 | 12 | 48398370 | COL2A1 | -0.04 | 9.5E-01 |  | 2.96 | 1.8E-03 |  | -0.15 | 8.1E-01 |  | 0.72 | 1.8E-01 |  | -0.12 | 8.2E-01 |
| cg07218487 | 12 | 49418559 | KMT2D | -1.00 | 1.8E-01 |  | -1.68 | 4.6E-02 |  | -1.70 | 8.0E-03 |  | -1.50 | 5.0E-03 |  | -1.51 | 4.0E-03 |
| cg05493841 | 12 | 70083096 | BEST3 | 1.70 | 5.2E-03 |  | 0.82 | 2.6E-01 |  | 0.41 | 4.6E-01 |  | 0.52 | 2.7E-01 |  | 0.90 | 4.1E-02 |
| cg12111758 | 12 | 111472375 | CUX2 | -1.45 | 3.3E-03 |  | -1.09 | 6.0E-02 |  | -0.05 | 9.1E-01 |  | -0.44 | 2.6E-01 |  | -0.69 | 5.8E-02 |
| cg05529091 | 12 | 111475875 | CUX2 | 1.32 | 7.8E-03 |  | 1.04 | 4.7E-02 |  | 0.49 | 2.7E-01 |  | 0.70 | 5.2E-02 |  | 0.81 | 2.5E-02 |
| cg13300744 | 12 | 111536664 | CUX2 | 0.49 | 5.3E-01 |  | 1.56 | 5.3E-02 |  | 1.60 | 1.2E-02 |  | 1.55 | 3.3E-03 |  | 1.18 | 2.6E-02 |
| cg20108695 | 12 | 111598328 | CUX2 | -2.71 | 6.1E-04 |  | -1.17 | 1.4E-01 |  | -0.84 | 1.9E-01 |  | -0.81 | 1.3E-01 |  | -1.48 | 6.1E-03 |
| cg04853995 | 12 | 112550460 | NAA25 | -0.02 | 9.7E-01 |  | -2.01 | 3.2E-03 |  | -0.77 | 1.6E-01 |  | -1.14 | 1.1E-02 |  | -0.43 | 3.3E-01 |
| cg04462561 | 13 | 24837937 | SPATA13 | -0.42 | 3.5E-01 |  | 1.02 | 3.7E-02 |  | 0.82 | 4.3E-02 |  | 0.89 | 7.2E-03 |  | 0.25 | 4.3E-01 |
| cg18951977 | 14 | 54421397 | BMP4 | 0.04 | 9.3E-01 |  | -2.01 | 4.9E-04 |  | 0.08 | 8.5E-01 |  | -0.64 | 7.3E-02 |  | 0.09 | 7.9E-01 |
| cg08169864 | 14 | 54421413 | BMP4 | 0.44 | 5.6E-01 |  | 0.62 | 4.2E-01 |  | 1.90 | 4.3E-03 |  | 1.22 | 1.9E-02 |  | 1.27 | 1.7E-02 |
| cg18298494 | 14 | 76447327 | TGFB3 | -2.23 | 8.6E-03 |  | -1.02 | 2.7E-01 |  | -1.17 | 9.4E-02 |  | -0.95 | 1.1E-01 |  | -1.53 | 8.6E-03 |
| cg20089531 | 14 | 92573973 | ATXN3 | 0.93 | 1.7E-01 |  | -1.84 | 9.4E-03 |  | 0.30 | 5.8E-01 |  | -0.33 | 4.6E-01 |  | 0.55 | 2.3E-01 |
| cg27137003 | 15 | 74831097 | ARID3B | 3.19 | 6.3E-04 |  | 1.78 | 5.7E-02 |  | 0.57 | 4.4E-01 |  | 0.83 | 1.7E-01 |  | 1.47 | 1.8E-02 |
| cg25506127 | 15 | 74907002 | CLK3 | -1.83 | 3.3E-03 |  | 1.08 | 1.6E-01 |  | -1.10 | 3.5E-02 |  | -0.28 | 5.3E-01 |  | -1.26 | 3.4E-03 |
| cg01370575 | 15 | 74914482 | CLK3 | -0.64 | 3.5E-01 |  | -2.78 | 7.5E-04 |  | -0.48 | 4.4E-01 |  | -1.22 | 2.2E-02 |  | -0.56 | 2.7E-01 |
| cg06979726 | 16 | 84851070 | CRISPLD2 | 1.51 | 6.4E-03 |  | 0.07 | 9.1E-01 |  | -0.02 | 9.7E-01 |  | -0.05 | 9.1E-01 |  | 0.54 | 1.5E-01 |
| cg05632908 | 16 | 84880339 | CRISPLD2 | -0.64 | 2.1E-01 |  | -1.87 | 1.8E-03 |  | -0.43 | 3.3E-01 |  | -0.91 | 1.5E-02 |  | -0.53 | 1.4E-01 |
| cg06250127 | 16 | 84940830 | CRISPLD2 | 0.51 | 3.4E-01 |  | -1.58 | 7.8E-03 |  | -0.10 | 8.3E-01 |  | -0.61 | 1.1E-01 |  | 0.16 | 6.8E-01 |
| cg10432440 | 16 | 86597802 | FOXC2 | -0.01 | 9.8E-01 |  | -0.21 | 6.6E-01 |  | -0.94 | 8.9E-03 |  | -0.65 | 3.6E-02 |  | -0.56 | 5.6E-02 |
| cg09360770 | 16 | 86600326 | FOXC2 | 0.04 | 9.5E-01 |  | 2.22 | 5.6E-03 |  | -0.67 | 2.3E-01 |  | 0.35 | 4.6E-01 |  | -0.27 | 5.6E-01 |
| cg23402144 | 17 | 9099485 | NTN1 | 0.32 | 6.2E-01 |  | 0.74 | 3.2E-01 |  | 1.65 | 4.9E-03 |  | 1.12 | 2.3E-02 |  | 1.09 | 1.9E-02 |
| cg13209613 | 17 | 44849789 | WNT3 | -0.55 | 3.0E-01 |  | -0.22 | 6.9E-01 |  | 1.16 | 8.6E-03 |  | 0.66 | 7.1E-02 |  | 0.51 | 1.6E-01 |
| cg20656587 | 17 | 44895317 | WNT3 | -0.89 | 7.2E-03 |  | -0.07 | 8.3E-01 |  | -0.04 | 8.7E-01 |  | 0.02 | 9.3E-01 |  | -0.37 | 9.5E-02 |
| cg27185128 | 17 | 45000622 | GOSR2 | -0.43 | 4.1E-01 |  | -1.11 | 3.2E-02 |  | -1.05 | 1.7E-02 |  | -1.10 | 2.9E-03 |  | -0.76 | 3.9E-02 |
| cg26076846 | 17 | 61368544 | TANC2 | -1.98 | 4.0E-03 |  | -1.77 | 2.6E-02 |  | -0.27 | 6.7E-01 |  | -0.63 | 2.3E-01 |  | -0.92 | 6.7E-02 |
| cg25520488 | 17 | 61499194 | TANC2 | -0.68 | 2.8E-01 |  | 1.64 | 2.2E-02 |  | 1.26 | 1.8E-02 |  | 1.33 | 3.3E-03 |  | 0.46 | 2.8E-01 |
| cg07102474 | 17 | 61499248 | TANC2 | -0.14 | 8.3E-01 |  | 1.92 | 7.6E-03 |  | -0.14 | 8.0E-01 |  | 0.70 | 1.3E-01 |  | -0.08 | 8.5E-01 |
| cg12484037 | 17 | 68165632 | KCNJ2 | -2.53 | 2.2E-04 |  | -0.43 | 5.4E-01 |  | -0.46 | 4.1E-01 |  | -0.49 | 2.9E-01 |  | -1.22 | 8.6E-03 |
| cg03547745 | 17 | 70117522 | SOX9 | 1.99 | 6.4E-03 |  | 0.58 | 4.3E-01 |  | 0.33 | 5.7E-01 |  | 0.35 | 4.6E-01 |  | 0.93 | 5.6E-02 |
| cg21434114 | 18 | 3450282 | TGIF1 | 1.63 | 2.6E-03 |  | 0.69 | 2.6E-01 |  | 0.11 | 8.1E-01 |  | 0.20 | 6.1E-01 |  | 0.66 | 8.0E-02 |
| cg10580691 | 18 | 3451079 | TGIF1 | -1.30 | 9.6E-03 |  | 0.34 | 5.6E-01 |  | -0.47 | 2.7E-01 |  | -0.25 | 5.0E-01 |  | -0.68 | 4.8E-02 |
| cg00033220 | 18 | 3451606 | TGIF1 | 1.48 | 4.5E-02 |  | 1.39 | 8.3E-02 |  | 1.49 | 1.5E-02 |  | 1.38 | 7.6E-03 |  | 1.40 | 5.2E-03 |
| cg11894504 | 19 | 2042391 | MKNK2 | 0.75 | 2.4E-01 |  | -1.71 | 1.2E-02 |  | -0.79 | 1.5E-01 |  | -1.24 | 7.4E-03 |  | -0.16 | 7.2E-01 |
| cg06521145 | 19 | 2048650 | MKNK2 | -0.63 | 5.4E-01 |  | -4.11 | 1.7E-03 |  | -0.03 | 9.8E-01 |  | -1.22 | 1.1E-01 |  | -0.03 | 9.6E-01 |
| cg14326260 | 20 | 3913389 | RNF24 | -0.90 | 1.9E-01 |  | -2.39 | 2.3E-03 |  | -1.37 | 2.5E-02 |  | -1.56 | 2.5E-03 |  | -1.09 | 2.6E-02 |
| cg23834525 | 20 | 3995385 | RNF24 | -0.19 | 7.5E-01 |  | 1.42 | 3.6E-02 |  | 1.06 | 5.6E-02 |  | 1.26 | 6.6E-03 |  | 0.48 | 2.6E-01 |
| cg03224175 | 20 | 3996790 | RNF24 | -2.16 | 3.6E-03 |  | -1.24 | 1.2E-01 |  | -0.39 | 5.5E-01 |  | -0.57 | 2.8E-01 |  | -1.13 | 2.7E-02 |
| cg00965748 | 20 | 39319235 | MAFB | 1.84 | 7.7E-03 |  | 1.75 | 1.9E-02 |  | 0.56 | 3.4E-01 |  | 0.99 | 4.2E-02 |  | 1.02 | 3.6E-02 |
| cg06682383 | 22 | 30275268 | MTMR3 | -1.69 | 7.6E-03 |  | 0.42 | 5.3E-01 |  | 0.26 | 6.4E-01 |  | 0.26 | 5.7E-01 |  | -0.55 | 2.2E-01 |
| cg21461927 | 22 | 36697043 | MYH9 | -0.50 | 5.1E-01 |  | 0.16 | 8.5E-01 |  | -2.07 | 2.0E-03 |  | -1.08 | 5.5E-02 |  | -1.55 | 4.4E-03 |
| cg09051119 | 22 | 36721648 | MYH9 | -0.96 | 1.8E-01 |  | 0.33 | 6.8E-01 |  | -2.34 | 2.3E-05 |  | -1.52 | 7.6E-04 |  | -1.72 | 9.9E-05 |
| cg20478080 | 22 | 45133246 | PRR5-ARHGAP8 | -0.07 | 9.1E-01 |  | -2.27 | 1.4E-03 |  | 0.81 | 9.7E-02 |  | -0.15 | 7.2E-01 |  | 0.47 | 2.4E-01 |
| cg06647930 | 22 | 45135965 | PRR5-ARHGAP8 | -1.12 | 1.8E-02 |  | -0.02 | 9.7E-01 |  | -0.91 | 3.6E-02 |  | -0.60 | 1.1E-01 |  | -0.92 | 8.1E-03 |
| cg21608237 | 22 | 45148229 | ARHGAP8 | 0.99 | 1.0E-01 |  | -1.76 | 8.6E-03 |  | 0.18 | 7.2E-01 |  | -0.41 | 3.3E-01 |  | 0.44 | 2.9E-01 |
| cg09685351 | 22 | 45148319 | ARHGAP8 | 0.44 | 2.8E-01 |  | 1.48 | 2.3E-03 |  | 0.72 | 5.1E-02 |  | 0.87 | 5.0E-03 |  | 0.61 | 4.1E-02 |
| cg05093999 | X | 10851789 | MID1 | 1.42 | 2.9E-03 |  | -0.23 | 6.8E-01 |  | -0.39 | 3.3E-01 |  | -0.31 | 3.8E-01 |  | 0.26 | 4.2E-01 |
| cg19790752 | X | 10852973 | MID1 | -0.32 | 4.2E-01 |  | 0.24 | 6.1E-01 |  | -0.95 | 6.6E-03 |  | -0.44 | 1.4E-01 |  | -0.63 | 2.4E-02 |
| cg15627188 | X | 40005337 | BCOR | -0.47 | 1.4E-01 |  | -1.27 | 8.5E-04 |  | -0.13 | 6.4E-01 |  | -0.49 | 3.8E-02 |  | -0.34 | 1.4E-01 |
| cg03140487 | X | 40027582 | BCOR | -1.95 | 9.9E-03 |  | -1.33 | 1.0E-01 |  | 0.14 | 8.4E-01 |  | -0.45 | 4.2E-01 |  | -0.60 | 2.5E-01 |
| cg09075063 | X | 40036659 | BCOR | 0.37 | 4.2E-01 |  | 0.71 | 1.4E-01 |  | 1.27 | 2.7E-03 |  | 0.94 | 5.7E-03 |  | 0.85 | 9.5E-03 |
| cg19840133 | X | 48754590 | PQBP1 | -0.77 | 3.0E-02 |  | -0.98 | 1.1E-02 |  | -0.65 | 4.0E-02 |  | -0.73 | 4.6E-03 |  | -0.59 | 2.2E-02 |
| cg26807389 | X | 48760712 | PQBP1 | 0.67 | 2.2E-01 |  | -0.11 | 8.6E-01 |  | 1.21 | 1.7E-02 |  | 0.66 | 1.0E-01 |  | 1.07 | 7.2E-03 |
| cg00839572 | X | 68047847 | EFNB1 | -0.23 | 6.2E-01 |  | -0.47 | 3.8E-01 |  | -1.21 | 4.5E-03 |  | -0.91 | 8.4E-03 |  | -0.79 | 1.9E-02 |
| cg09143059 | X | 153585715 | FLNA | -0.64 | 1.9E-01 |  | -1.56 | 6.2E-03 |  | -0.86 | 4.1E-02 |  | -1.05 | 3.1E-03 |  | -0.73 | 3.1E-02 |
| cg01817230 | X | 153594995 | FLNA | -0.13 | 8.3E-01 |  | -1.02 | 1.4E-01 |  | -1.52 | 5.2E-03 |  | -1.39 | 2.0E-03 |  | -0.85 | 4.6E-02 |

**Table S6.** Differentially methylated regions (DMR) with Sidak multiple testing corrected P value <0.05

**Table S7.** CpGs and association results within Differentially methylated regions.
